# Mesenchymal VEGFA induces aberrant differentiation in heterotopic ossification

**DOI:** 10.1038/s41413-019-0075-6

**Published:** 2019-12-10

**Authors:** Charles Hwang, Simone Marini, Amanda K. Huber, David M. Stepien, Michael Sorkin, Shawn Loder, Chase A. Pagani, John Li, Noelle D. Visser, Kaetlin Vasquez, Mohamed A. Garada, Shuli Li, Jiajia Xu, Ching-Yun Hsu, Paul B. Yu, Aaron W. James, Yuji Mishina, Shailesh Agarwal, Jun Li, Benjamin Levi

**Affiliations:** 1 Department of Surgery, University of Michigan, Ann Arbor, MI 48109 USA; 2 Department of Computational Medicine and Bioinformatics, University of Michigan, Ann Arbor, MI 48109 USA; 3 Department of Pathology, Johns Hopkins University, Baltimore, MD 21218 USA; 4 Division of Cardiovascular Medicine, Department of Medicine, Brigham and Women’s Hospital, Harvard Medical School, Boston, MA 02115 USA; 5 Department of Biologic and Materials Sciences & Prosthodontics, School of Dentistry, University of Michigan, Ann Arbor, MI 48109 USA

**Keywords:** Bone, Pathogenesis

## Abstract

Heterotopic ossification (HO) is a debilitating condition characterized by the pathologic formation of ectopic bone. HO occurs commonly following orthopedic surgeries, burns, and neurologic injuries. While surgical excision may provide palliation, the procedure is often burdened with significant intra-operative blood loss due to a more robust contribution of blood supply to the pathologic bone than to native bone. Based on these clinical observations, we set out to examine the role of vascular signaling in HO. Vascular endothelial growth factor A (VEGFA) has previously been shown to be a crucial pro-angiogenic and pro-osteogenic cue during normal bone development and homeostasis. Our findings, using a validated mouse model of HO, demonstrate that HO lesions are highly vascular, and that VEGFA is critical to ectopic bone formation, despite lacking a contribution of endothelial cells within the developing anlagen.

## Introduction

Pathology from excess ectopic bone formation following musculoskeletal injury, or trauma-induced heterotopic ossification (HO), presents a substantial barrier to recovery in 20% of patients with hip replacements, musculoskeletal trauma, neurologic injury, and blast/burn injuries.^1,2^ Patients with HO experience substantive morbidity including chronic pain, restricted joint function, and open wounds. Effective therapeutics are lacking in contemporary medicine aside from surgical excision. However, these procedures can fail to completely reverse the joint contractures and restricted range of motion.^3^ Furthermore, even after a successful excision procedure, recurrence may occur via similar cellular mechanisms as observed in HO from the incident trauma.^4–6^

While the etiologies of HO are diverse, it often follows a predictable progression of endochondral ossification^7–10^ after acute inflammatory insult with proliferation of progenitor populations of multipotent mesenchymal stromal cells (MSCs),^11–13^ chondrogenesis, angiogenesis, and ossification^7^ resulting in highly vascular regions surrounding the HO.^14^ These observations parallel programming seen in normal developing bone and fracture healing, suggesting overlapping pathways between developing bone and posttraumatic HO such as the intimate coupling of vascular invasion and osteogenesis.^15^ Previous studies have further demonstrated MSCs differentiating into a cartilaginous scaffold and supportive matrix,^16^ forming a hypoxic environment responsible for the upregulation of HIF1a and VEGFA.^17–19^ During normal bone formation, VEGFA facilitates co-invasion of this anlagen by neovasculature and Osterix(Osx)-expressing osteoblast precursors,^16^ with increased delivery of oxygen to support growing metabolic activity.^20^ Similar migratory behavior to primary ossification centers by osteoblast precursors was observed in response to VEGFA secreted by chondrocytes.^21^ Disruption of these processes lead to impaired endochondral ossification.^8,22^ Notably, mice with conditional deletion of VEGFA in *Col2a* expressing cells demonstrate aberrations in cartilage development and increased chondrocyte apoptosis.^13,23^ The role of VEGFA as a pro-angiogenic/osteogenic signal^24–26^ fundamental to bone development and homeostasis^27^ highlights a promising candidate for uncoupling acute injury and ectopic bone formation. Though previous studies have demonstrated the role of endothelial progenitors and VEGFA^28^ in bone development and in a nontraumatic HO model, previous work did not identify the role of mesenchymal-derived VEGFA in traumatic HO.

Acute posttraumatic inflammation stimulates local^13,29^ and recruited MSCs to secrete a collection of soluble factors^30,31^ including VEGFA.^32^ However, MSCs have been well demonstrated to be a heterogeneous population^13^ and thus the true lineage and source of these progenitor cells have remained unclear. Various origins have been proposed for the progenitor cells responsible for HO including Tie2/vWF/VeCadherin-positive endothelium,^33^ Prx1/Mx-1/PDGFRα-positive muscle interstitial fibro-adipogenic progenitors,^34–36^ scleraxis (Scx) lineage tendon derived stem cells^37^ and a myriad of other identifying markers.^13,38–41^ Further illustrating the vast heterogeneity of involved cell lineages, additional peripheral progenitor niches such as those marked by odd-skipped related (Osr1, Osr2) genes^42^ and engrailed1 (En1)^43^ may also be involved although not yet implicated with HO.

Given the role of VEGFA during normal bone development and the infiltration of mesenchymal stem cells throughout the formation of HO, we hypothesized that interruption of progenitor-cell-derived VEGFA would lead to a reduction in posttraumatic HO formation. We demonstrate that mesenchymal progenitors are a significant source of VEGFA important for ectopic bone formation and cell-specific inhibition of this signal significantly reduces posttraumatic HO.

## Results

### Extremity trauma and inflammation induces a pro-angiogenic environment characterized by an increase in total endothelial structures

**Fig. 1:**
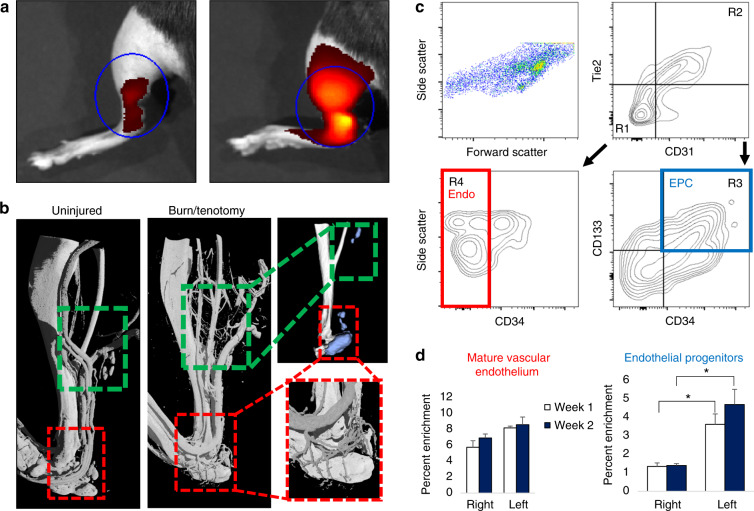
Focal injury superimposed upon systemic inflammation induces robust local angiogenesis and produces enrichment of endothelial progenitors at the injury site. **a** Angiosense IVIS demonstrates increased perfusion to injured hindlimb at 20 h post surgery (representative images from *n* = 3 per group). **b** MICROFIL perfusion nano-CT images and micro-CT of mature mice show networks of nascent vessel outgrowth in regions of subsequent HO formation following burn tenotomy (green inset) at 9 weeks. Vessels in the distal hindlimb exhibit dense vascular infiltration (red inset). **c** FACS gating schema of injury site 2 weeks after burn tenotomy. CD31^−^ Tie2^−^ CD34^+^ CD133^+^ endothelial progenitor cells (EPC, blue) identified via R1 + R3. CD31^+^ Tie2^+^ CD34^−^ mature endothelial cells (red) identified via R2 + R4. Remaining live events were collected by surface marker depletion. **d** Proportion of gated EPC and mature endothelium from all live cells as assessed by flow cytometry was quantified at 1 week and 2 weeks post burn tenotomy

Previous studies have demonstrated the upregulation of vascular signaling prior to pre-HO chondrogenesis.^28^ However, high-resolution imaging of the actual vessels have not been obtained in relation to HO after trauma. To do this, we performed multiple modalities to corroborate the increase in vascular density at multiple timepoints across the progression of endochondral ossification, including the acute phase after injury (20 h) and plateau of ossification (9 weeks). First, we used near infrared in vivo interrogation using Angiosense 750EX to image the mouse hindlimb and demonstrate a robust increase in blood flow (Fig. 1a). Next, to obtain a high resolution and anatomic assessment of the vascular network implicated with HO formation, we performed microfil injection and nano-CT imaging. We visualized two distinct regions of increased vascular growth around the calcaneus in the distal hindlimb (Fig. 1b, red inset) and proximal to the tibial/fibular fuse point in the injured leg. HO formed in close proximity to corresponding vascular beds at 9 weeks (as demonstrated by microCT analyzed at density thresholds above the intravascular polymer) leading us to conclude that there was indeed involvement of vasculature following the peripheral injury with both increased blood flow and angiogenesis at the injury site. To further characterize the cell surface markers of the infiltrating cells at the HO site at more subacute timepoints, we performed flow cytometry of the injured tissue, specifically interrogating two specific cell types: potentially increasing endothelium per microfil imaging and a progenitor niche capable of undergoing transition to connective tissues^44^ to contribute to the HO anlagen. FACS analysis of the injury site highlighted a significant enrichment of CD31^−^ Tie2^−^ CD34^+^ CD133^+ 45–47^ (Fig. 1c) endothelial progenitor cells (EPCs) in burn/tenotomy mice at 1 and 2 weeks (2.70 and 3.36-fold increases compared with uninjured contralateral leg, *P* = 0.032 and 0.002, respectively). There was not similar enrichment of mature endothelium at the 1 or 2 week timepoints (Fig. 1d). Given previous literature demonstrating the autocrine nature of VEGFA in vascular homeostasis,^48^ this cellular enrichment of EPCs is consistent with a robust induction of neovasculature and also suggest these progenitor cells as a possible source for VEGFA signaling involved in the angiogenesis associated with HO development consistent with previously described nontrauma HO models.^28^ Thus, we demonstrate using several different modalities that during HO formation, a rich, patent vascular network intimately surrounds the bone, is more prominent than in the uninjured extremity, and likely a facilitative influence in the progression of ectopic of endochondral ossification.

### Vascular progenitors are not the contributing source for VEGFA at the burn/tenotomy injury site

**Fig. 2:**
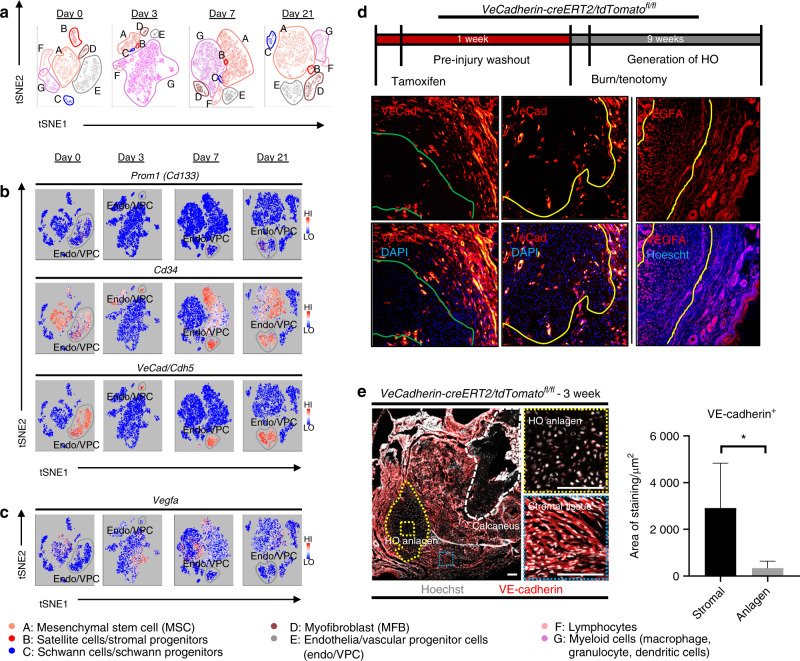
Vascular progenitors are not the source for VEGFA in HO formation. **a** Resultant seven clusters identifying cellular subpopulations within HO anlagen at respective timepoints. **b** Expression profiles for vascular progenitor genes *Prom1 (Cd133), CD34*, and *Vecad/Cdh5*. **c** Expression profile for *Vegfa* **d** Schematic for induction protocol of endogenous reporter mice. VeCadherin-creERT2/tdTomato^*fl/fl*^ identify cells with endothelial programming within outlined regions. Sections taken from distal hindlimb of injury. Right: Sections of injury site from wild-type 3 week burn/tenotomy mice labeled for VEGFA. **e** Tiled immunofluorescent micrograph of 3 week burn/tenotomy injury site in endogenous VeCadherin reporters. Quantifications for thresholded total positive area (FIJI/ImageJ) were performed across biological triplicate, with 2 hpf per specimen for both HO anlagen site vs. surrounding stroma (*n* = 6/group)

Given the enrichment of EPCs demonstrated by flow cytometry, we performed a directed single-cell RNA sequencing experiment in order to characterize transcriptome-level expression of *Vegfa* and the cellular subpopulations producing these transcripts at baseline preinjury (day 0), and days 3, 7, and 21 postinductive burn/tenotomy. Clustering analysis was performed by blinded biostatistician. In order to characterize the perturbation from baseline caused by inductive burn/tenotomy, clusters were first preliminarily generated unsupervised, and subsequently rank correlated by centroids. This allowed to consolidate clusters into seven cell families, and subsequently compare them across replicates both within a single timepoint and across all timepoints. Clusters show high intra-timepoint stability, as demonstrated by the red diagonals in rank correlation heatmaps for each timepoint and PCA plots. The only notable exception to correlation with self was cluster A that showed a reciprocal minor correlation with cluster D. Based on this high fidelity, replicates within timepoints were pooled for subsequent analysis, with proportional contribution to each cluster from all replicates confirmed by PCA plots (Supplemental Figs. 1–12). The resulting seven clusters (Fig. 2a) were identified via gene ontology terms and KEGG pathway analyses (Tables 1–7) in addition to characteristic gene expression (Supplemental Figs. 13–15): mesenchymal stem cells (*Pdgfra*), satellite cells with putative stromal progenitors (*Pax7*), Schwann cells/neural progenitors (*Plp1, Cnp, Dhh*), myofibroblasts (MFB) encompassing pericytes and smooth muscle cells (*Acta2, Mylk*), endothelia/vascular progenitor cells (*Cdh5, Pecam, Cd34*), lymphocytes (*Ms4a1*), and myeloid cells including macrophages (*Ccr2, Mrc1, Fcgr1*), granulocyte (*Ccr1, S100a9*), dendritic cells (*Cd209a*). Fold change and adjusted *P* values for genes of interest are included in Table 8. Notably, the endothelial/vascular progenitors cluster (D) demonstrated expression of both *Prom1(Cd133)* and *Cd34* (Fig. 2b, top/middle), corresponding to the EPC subpopulation identified in flow cytometry (Fig. 1c, d). This cluster also demonstrated high expression of mature endothelium genes (Supplemental Fig. 1) suggesting a mixed phenotype of the EPC and endothelium identified earlier in the flow cytometry. *VeCadherin*/*Cdh5* (Fig. 2b, bottom) also robustly marked this cellular subpopulation. However, this cluster did not exhibit high *Vegfa* expression levels (Fig. 2c) across any timepoints, suggesting these cells actually were not the likely source for VEGFA signal. To verify these findings, we looked to reconcile data from previous studies that have identified cells of the endothelial lineage to serve as potential HO progenitor cells using a Tie-Cre^33^ marker. These models, however, did not use inducible systems which allow for adult labeling of cells, which is necessary to assess the true contribution of adult endothelial cells to posttraumatic HO. To address this limitation, inducible tdTomato reporter mice expressing creERT driven by the Cdh5 promoter were treated with tamoxifen with washout prior to burn/tenotomy to interrogate the spatial arrangement of early cells of endothelial lineage. Histological analysis demonstrated Cdh5+ cells spatially peripheral to the forming HO lesion but a distinct absence of Cdh5+ cells in the inflamed soft tissue deep to the cutaneous/stromal layers at 1 week (Fig. 2d, green). These cells remained a minor population in the progressing HO anlagen even at the 3 week timepoint (Fig. 2d, yellow), in stark contrast to the copious number of VEGFA-expressing cells seen in the same region (Fig. 2d, right). With the observation of Cdh5 enrichment in the periphery of pre-HO anlagen, we corroborated the significant enrichment in the surrounding stroma compared with pre-HO tissue via thresholded quantification of endogenous reporter micrographs (Fig. 2e). Thus, we verified that progenitors from endothelial lineages were not likely candidate precursors to the formation of HO, nor sources for VEGFA within the HO niche. The relatively few numbers of Cdh5+ cells within the HO analgen is consistent with previous studies showing that mesenchymal cells of nonendothelial lineage (i.e. *Prx*+, *Mx1*+, or *Scx*+) are the traumatic HO progenitors.^34,36,37,49^ Having demonstrated that HO lesions are highly vascular but that cells of endothelial origin do not densely populate the interior of the HO anlagen, we next examined whether VEGFA expression spatially correlated with other cellular subpopulations.

**Table 1:** Cluster A: Top 10 upregulated terms from KEGG and Gene Ontology Domains

ID	Name	Concept	#Genes	FDR
mmu04141	Protein processing in endoplasmic reticulum	KEGG	156	5.56E−10
mmu00510	N-Glycan biosynthesis	KEGG	43	1.79E−06
mmu04512	ECM-receptor interaction	KEGG	82	1.95E−05
mmu03060	Protein export	KEGG	22	7.76E−05
mmu04310	Wnt signaling pathway	KEGG	137	0.000 162
mmu00532	Glycosaminoglycan biosynthesis - chondroitin sulfate/dermatan sulfate	KEGG	21	0.000 213 33
mmu00534	Glycosaminoglycan biosynthesis - heparan sulfate/heparin	KEGG	22	0.000 468 57
mmu04120	Ubiquitin mediated proteolysis	KEGG	127	0.001 014 98
mmu05217	Basal cell carcinoma	KEGG	50	0.001 937 43
mmu04350	TGF-beta signaling pathway	KEGG	78	0.005 932 52
GO:0005578	Proteinaceous extracellular matrix	GOCC	284	2.14E−36
GO:0031012	Extracellular matrix	GOCC	410	1.73E−27
GO:0044420	Extracellular matrix component	GOCC	121	9.66E−17
GO:0005783	Endoplasmic reticulum	GOCC	1 234	1.58E−13
GO:0005794	Golgi apparatus	GOCC	1 113	1.57E−12
GO:0005604	Basement membrane	GOCC	93	3.48E−09
GO:0044432	Endoplasmic reticulum part	GOCC	423	2.29E−07
GO:0005793	Endoplasmic reticulum-Golgi intermediate compartment	GOCC	53	3.12E−07
GO:0005581	Collagen trimer	GOCC	71	4.70E−06
GO:0005801	*Cis*-golgi network	GOCC	32	4.70E−06
GO:0005201	Extracellular matrix structural constituent	GOMF	35	5.06E−07
GO:0005539	Glycosaminoglycan binding	GOMF	157	1.17E−06
GO:0008201	Heparin Binding	GOMF	120	1.25E−06
GO:0016758	Transferase activity, transferring hexosyl groups	GOMF	149	2.51E−06
GO:1901681	Sulfur compound binding	GOMF	183	5.05E−05
GO:0019838	Growth factor binding	GOMF	116	0.000 216 25
GO:0016757	Transferase activity, transferring glycosyl groups	GOMF	229	0.000 429 31
GO:0017147	Wnt-protein binding	GOMF	30	0.000 451 6
GO:0004576	Oligosaccharyl transferase activity	GOMF	57	0.000 524 88
GO:0001968	Fibronectin binding	GOMF	27	0.000 738 19
GO:0001501	Skeletal system development	GOBP	412	3.26E−15
GO:0051216	Cartilage development	GOBP	151	1.10E−14
GO:0061448	Connective tissue development	GOBP	205	4.24E−14
GO:0030198	Extracellular matrix organization	GOBP	189	1.43E−12
GO:0043062	Extracellular structure organization	GOBP	190	1.94E−12
GO:0009100	Glycoprotein metabolic process	GOBP	265	8.91E−12
GO:0048736	Appendage development	GOBP	160	2.40E−11
GO:0060173	Limb development	GOBP	160	2.40E−11
GO:0035107	Appendage morphogenesis	GOBP	142	7.77E−11
GO:0035108	Limb morphogenesis	GOBP	142	7.77E−11

**Table 2:** Cluster B: Top 10 upregulated terms from KEGG and Gene Ontology Domains

ID	Name	Concept	#Genes	FDR
mmu03010	Ribosome	KEGG	68	2.00E−19
mmu05412	Arrhythmogenic right ventricular cardiomyopathy (ARVC)	KEGG	71	0.000 437 49
mmu04080	Neuroactive ligand-receptor interaction	KEGG	154	0.017 800 51
mmu04740	Olfactory transduction	KEGG	21	0.028 569 4
mmu05410	Hypertrophic cardiomyopathy (HCM)	KEGG	77	0.093 835 47
mmu05414	Dilated cardiomyopathy	KEGG	80	0.106 303 39
mmu04020	Calcium signaling pathway	KEGG	143	0.159 608 01
mmu04260	Cardiac muscle contraction	KEGG	59	0.179 346 27
mmu04960	Aldosterone-regulated sodium reabsorption	KEGG	35	0.185 410 25
mmu04340	Hedgehog signaling pathway	KEGG	45	0.230 030 64
GO:0022626	Cytosolic ribosome	GOCC	80	1.22E−38
GO:0044391	Ribosomal subunit	GOCC	131	5.61E−31
GO:0022625	Cytosolic large ribosomal subunit	GOCC	43	5.86E−28
GO:0044445	Cytosolic part	GOCC	163	3.82E−27
GO:0005840	Ribosome	GOCC	180	3.30E−26
GO:0015934	Large ribosomal subunit	GOCC	76	2.36E−21
GO:0030016	Myofibril	GOCC	167	7.33E−21
GO:0043292	Contractile fiber	GOCC	180	8.64E−19
GO:0030017	Sarcomere	GOCC	148	1.09E−18
GO:0044449	Contractile fiber part	GOCC	161	1.68E−18
GO:0003735	Structural constituent of ribosome	GOMF	151	3.61E−26
GO:0019843	rRNA binding	GOMF	57	2.47E−14
GO:0005198	Structural molecule activity	GOMF	432	1.66E−13
GO:0000981	RNA polymerase II transcription factor activity, sequence-specific DNA binding	GOMF	503	7.69E−09
GO:0001071	Nucleic acid binding transcription factor activity	GOMF	804	1.48E−07
GO:0003700	Transcription factor activity, sequence-specific DNA binding	GOMF	804	1.48E−07
GO:0030552	cAMP binding	GOMF	21	1.60E−07
GO:0001228	Transcriptional activator activity, RNA polymerase II transcription regulatory region sequence-specific binding	GOMF	263	7.22E−06
GO:0043565	Sequence-specific DNA binding	GOMF	769	7.64E−06
GO:0030551	Cyclic nucleotide binding	GOMF	31	8.48E−06
GO:0007517	Muscle organ development	GOBP	307	1.56E−22
GO:0007519	Skeletal Muscle tissue development	GOBP	149	2.23E−21
GO:0060538	Skeletal muscle organ development	GOBP	153	3.10E−21
GO:0014706	Striated muscle tissue development	GOBP	329	1.84E−20
GO:0060537	Muscle tissue development	GOBP	344	4.68E−20
GO:0061061	Muscle structure development	GOBP	527	1.75E−18
GO:0035914	Skeletal muscle cell differentiation	GOBP	66	8.23E−18
GO:0060415	Muscle tissue morphogenesis	GOBP	66	1.76E−15
GO:0055001	Muscle cell development	GOBP	149	3.30E−15
GO:0006936	Muscle contraction	GOBP	226	7.59E−15

**Table 3:** Cluster C: Top 10 upregulated terms from KEGG and Gene Ontology Domains

ID	Name	Concept type	X.Genes	FDR
mmu04514	Cell adhesion molecules (CAMs)	KEGG	108	4.99E−07
mmu04360	Axon guidance	KEGG	123	7.75E−06
mmu05412	Arrhythmogenic right ventricular cardiomyopathy (ARVC)	KEGG	69	0.003 312 12
mmu04530	Tight junction	KEGG	107	0.005 248 1
mmu04971	Gastric acid secretion	KEGG	59	0.027 180 8
mmu04012	ErbB signaling pathway	KEGG	84	0.040 082 96
mmu04512	ECM-receptor interaction	KEGG	81	0.048 468 92
mmu05414	Dilated cardiomyopathy	KEGG	78	0.048 468 92
mmu05410	Hypertrophic cardiomyopathy (HCM)	KEGG	74	0.081 685 01
mmu04540	Gap junction	KEGG	75	0.081 685 01
GO:0030424	Axon	GOCC	435	2.03E−23
GO:0097458	Neuron Part	GOCC	1 223	8.88E−20
GO:0043005	Neuron projection	GOCC	963	4.47E−18
GO:0043209	Myelin sheath	GOCC	195	7.26E−18
GO:0045202	Synapse	GOCC	678	2.59E−14
GO:0033267	Axon part	GOCC	232	1.12E−13
GO:0044304	Main axon	GOCC	65	2.20E−12
GO:0036477	Somatodendritic compartment	GOCC	700	3.91E−11
GO:0044456	Synapse part	GOCC	510	3.91E−11
GO:0043218	Compact myelin	GOCC	19	5.42E−11
GO:0005249	Voltage-gated potassium channel activity	GOMF	55	2.78E−07
GO:0005267	Potassium channel activity	GOMF	80	2.36E−06
GO:0008092	Cytoskeletal protein binding	GOMF	711	4.23E−06
GO:0015079	Potassium ion transmembrane transporter activity	GOMF	107	4.23E−06
GO:0005200	Structural constituent of cytoskeleton	GOMF	60	1.22E−05
GO:0015267	Channel activity	GOMF	293	1.24E−05
GO:0022803	Passive transmembrane transporter activity	GOMF	293	1.24E−05
GO:0005251	Delayed rectifier potassium channel activity	GOMF	23	4.27E−05
GO:0022836	Gated channel activity	GOMF	199	0.000 110 22
GO:0005216	Ion Channel activity	GOMF	264	0.000 123 54
GO:0010001	Glial Cell differentiation	GOBP	174	1.23E−33
GO:0007272	Ensheathment of neurons	GOBP	108	2.31E−30
GO:0008366	Axon ensheathment	GOBP	108	2.31E−30
GO:0042552	Myelination	GOBP	106	6.00E−30
GO:0042063	Gliogenesis	GOBP	232	2.94E−28
GO:0022008	Neurogenesis	GOBP	1 289	6.92E−23
GO:0021782	Glial Cell development	GOBP	75	4.85E−17
GO:0048699	Generation of neurons	GOBP	1 198	2.86E−16
GO:0014037	Schwann cell differentiation	GOBP	32	4.24E−16
GO:0007422	Peripheral nervous system development	GOBP	63	1.41E−15

**Table 4:** Cluster D: Top 10 upregulated terms from KEGG and Gene Ontology Domains

ID	Name	Concept type	X.Genes	FDR
mmu04270	Vascular smooth muscle contraction	KEGG	91	6.51E−17
mmu05412	Arrhythmogenic right ventricular cardiomyopathy (ARVC)	KEGG	70	7.57E−11
mmu04510	Focal adhesion	KEGG	192	6.84E−10
mmu05414	Dilated cardiomyopathy	KEGG	79	1.51E−09
mmu05410	Hypertrophic cardiomyopathy (HCM)	KEGG	75	2.08E−08
mmu04260	Cardiac muscle contraction	KEGG	58	2.13E−07
mmu04020	Calcium signaling pathway	KEGG	145	7.88E−07
mmu04512	ECM-receptor interaction	KEGG	81	6.03E−06
mmu05012	Parkinson's disease	KEGG	102	1.53E−05
mmu04970	Salivary secretion	KEGG	57	0.000 526 44
GO:0043292	Contractile fiber	GOCC	178	2.54E−24
GO:0030016	Myofibril	GOCC	165	6.97E−24
GO:0044449	Contractile fiber part	GOCC	159	6.97E−24
GO:0042383	Sarcolemma	GOCC	124	8.03E−24
GO:0030018	Z disc	GOCC	89	1.06E−22
GO:0030017	Sarcomere	GOCC	146	1.29E−21
GO:0031674	I band	GOCC	101	9.96E−21
GO:0032432	Actin filament bundle	GOCC	57	7.27E−17
GO:0030055	Cell-substrate junction	GOCC	342	2.42E−15
GO:0005924	Cell-substrate adherens junction	GOCC	338	1.18E−14
GO:0005261	Cation channel activity	GOMF	204	1.07E−07
GO:0008324	Cation transmembrane transporter activity	GOMF	416	1.72E−07
GO:0022890	inorganic cation transmembrane transporter activity	GOMF	362	1.83E−07
GO:0003779	Actin binding	GOMF	326	4.05E−07
GO:0046873	Metal ion transmembrane transporter activity	GOMF	296	1.05E−06
GO:0022836	Gated channel activity	GOMF	205	5.27E−06
GO:0005216	Ion Channel activity	GOMF	272	5.35E−06
GO:0030551	Cyclic nucleotide binding	GOMF	32	6.42E−06
GO:0015075	Ion transmembrane transporter activity	GOMF	538	8.49E−06
GO:0008092	Cytoskeletal protein binding	GOMF	714	1.18E−05
GO:0003012	Muscle system process	GOBP	293	5.28E−32
GO:0006936	Muscle contraction	GOBP	224	5.28E−32
GO:1903522	Regulation of blood circulation	GOBP	190	2.05E−24
GO:0008015	Blood circulation	GOBP	347	1.16E−21
GO:0003013	Circulatory system process	GOBP	351	1.99E−21
GO:0008016	Regulation of heart contraction	GOBP	125	2.07E−21
GO:0061061	Muscle structure development	GOBP	527	1.46E−20
GO:0003008	System process	GOBP	1 104	2.91E−20
GO:0044057	Regulation of system process	GOBP	382	7.31E−20
GO:0006939	Smooth muscle contraction	GOBP	88	2.35E−18

**Table 5:** Cluster E: Top 10 upregulated terms from KEGG and Gene Ontology Domains

ID	Name	Concept	#Genes	FDR
mmu04360	Axon guidance	KEGG	122	1.99E−10
mmu04530	Tight junction	KEGG	108	4.12E−08
mmu04670	Leukocyte transendothelial migration	KEGG	100	3.37E−05
mmu04270	Vascular smooth muscle contraction	KEGG	91	0.001 949 41
mmu04070	Phosphatidylinositol signaling system	KEGG	70	0.002 174 78
mmu05216	Thyroid cancer	KEGG	29	0.004 564 17
mmu04520	Adherens junction	KEGG	67	0.005 146 28
mmu04720	Long-term potentiation	KEGG	62	0.011 450 77
mmu05100	Bacterial invasion of epithelial cells	KEGG	68	0.012 868 56
mmu04330	Notch signaling pathway	KEGG	46	0.013 308 21
GO:0030054	Cell Junction	GOCC	1 174	5.40E−17
GO:0005911	Cell–cell junction	GOCC	570	1.39E−15
GO:0070161	Anchoring junction	GOCC	609	1.84E−13
GO:0005912	Adherens junction	GOCC	596	1.94E−13
GO:0005913	Cell–cell adherens junction	GOCC	302	3.22E−11
GO:0042641	Actomyosin	GOCC	61	1.16E−09
GO:0030027	Lamellipodium	GOCC	136	2.50E−09
GO:0098590	Plasma Membrane region	GOCC	698	5.05E−09
GO:0001725	Stress fiber	GOCC	52	5.68E−09
GO:0032432	Actin Filament bundle	GOCC	57	5.68E−09
GO:0045296	Cadherin binding	GOMF	281	5.00E−10
GO:0098641	Cadherin binding involved in cell-cell adhesion	GOMF	262	5.71E−08
GO:0098631	Protein binding involved in cell adhesion	GOMF	274	6.95E−08
GO:0098632	Protein binding involved in cell-cell adhesion	GOMF	269	8.48E−08
GO:0050839	Cell Adhesion molecule binding	GOMF	395	4.10E−07
GO:0005088	Ras guanyl-nucleotide exchange factor activity	GOMF	107	5.84E−06
GO:0005112	Notch binding	GOMF	16	1.49E−05
GO:0019904	Protein domain specific binding	GOMF	599	4.60E−05
GO:0005085	Guanyl-nucleotide exchange factor activity	GOMF	169	5.78E−05
GO:0001071	Nucleic acid binding transcription factor activity	GOMF	806	0.000 465 87
GO:0003158	Endothelium development	GOBP	99	1.22E−20
GO:0045446	Endothelial cell differentiation	GOBP	85	1.06E−19
GO:0001525	Angiogenesis	GOBP	386	4.52E−17
GO:0001667	Ameboidal-type cell migration	GOBP	306	1.39E−16
GO:0043542	Endothelial cell migration	GOBP	137	4.28E−16
GO:0048514	Blood Vessel morphogenesis	GOBP	467	4.29E−16
GO:0072358	Cardiovascular system development	GOBP	587	1.10E−15
GO:0001944	Vasculature development	GOBP	576	2.60E−15
GO:0001568	Blood vessel development	GOBP	552	3.65E−15
GO:0001885	Endothelial cell development	GOBP	53	5.44E−15

**Table 6:** Cluster F: Top 10 upregulated terms from KEGG and Gene Ontology Domains

ID	Name	Concept	#Genes	FDR
mmu03010	Ribosome	KEGG	68	3.07E−25
mmu05340	Primary immunodeficiency	KEGG	31	3.98E−19
mmu04660	T-cell receptor signaling pathway	KEGG	105	1.28E−16
mmu05332	Graft-versus-host disease	KEGG	28	4.22E−15
mmu05320	Autoimmune thyroid disease	KEGG	29	4.18E−14
mmu05330	Allograft rejection	KEGG	30	1.33E−13
mmu04650	Natural killer cell mediated cytotoxicity	KEGG	92	2.20E−13
mmu04940	Type I diabetes mellitus	KEGG	33	3.65E−11
mmu04640	Hematopoietic cell lineage	KEGG	71	1.84E−10
mmu04060	Cytokine-cytokine receptor interaction	KEGG	199	8.44E−10
GO:0022626	Cytosolic Ribosome	GOCC	80	2.70E−31
GO:0009897	External side of plasma membrane	GOCC	228	1.65E−28
GO:0098552	Side of membrane	GOCC	391	1.20E−27
GO:0044391	Ribosomal subunit	GOCC	131	1.01E−22
GO:0001772	Immunological synapse	GOCC	32	2.26E−21
GO:0044445	Cytosolic part	GOCC	163	1.36E−20
GO:0022625	Cytosolic large ribosomal subunit	GOCC	43	9.01E−20
GO:0005840	Ribosome	GOCC	179	1.35E−17
GO:0022627	Cytosolic small ribosomal subunit	GOCC	30	1.19E−15
GO:0015934	Large ribosomal subunit	GOCC	76	1.88E−13
GO:0003735	Structural constituent of ribosome	GOMF	151	3.26E−19
GO:0005070	SH3/SH2 adapter activity	GOMF	23	3.78E−12
GO:0003823	Antigen binding	GOMF	51	4.75E−12
GO:0004896	Cytokine receptor activity	GOMF	80	9.70E−12
GO:0004872	Receptor activity	GOMF	772	3.96E−11
GO:0004888	Transmembrane signaling receptor activity	GOMF	545	3.96E−11
GO:0038023	Signaling receptor activity	GOMF	626	3.96E−11
GO:0060089	Molecular transducer activity	GOMF	772	3.96E−11
GO:0099600	Transmembrane receptor activity	GOMF	568	5.16E−11
GO:0019843	rRNA binding	GOMF	57	3.40E−10
GO:0046649	Lymphocyte activation	GOBP	538	5.60E−48
GO:0045321	Leukocyte activation	GOBP	629	6.12E−44
GO:0042110	T-cell activation	GOBP	381	1.97E−42
GO:0070489	T-cell aggregation	GOBP	381	1.97E−42
GO:0071593	Lymphocyte aggregation	GOBP	382	1.97E−42
GO:0070486	Leukocyte aggregation	GOBP	388	5.58E−42
GO:0007159	Leukocyte cell-cell adhesion	GOBP	414	1.40E−41
GO:0030098	Lymphocyte differentiation	GOBP	294	9.69E−38
GO:0001775	Cell activation	GOBP	726	5.47E−37
GO:0030217	T-cell differentiation	GOBP	218	2.00E−36

**Table 7:** Cluster G: Top 10 upregulated terms from KEGG and Gene Ontology Domains

ID	Name	Concept	#Genes	FDR
mmu04142	Lysosome	KEGG	119	5.08E−14
mmu04145	Phagosome	KEGG	128	6.29E−13
mmu05140	Leishmaniasis	KEGG	63	6.29E−13
mmu05150	Staphylococcus aureus infection	KEGG	42	7.05E−10
mmu05323	Rheumatoid arthritis	KEGG	74	6.01E−09
mmu04062	Chemokine signaling pathway	KEGG	164	1.71E−08
mmu04380	Osteoclast differentiation	KEGG	111	3.92E−08
mmu00190	Oxidative phosphorylation	KEGG	107	5.01E−08
mmu04612	Antigen processing and presentation	KEGG	53	5.60E−08
mmu04620	Toll-like receptor signaling pathway	KEGG	86	6.44E−08
GO:0000323	Lytic Vacuole	GOCC	383	2.20E−27
GO:0005764	Lysosome	GOCC	383	2.20E−27
GO:0005773	Vacuole	GOCC	910	2.20E−16
GO:0098552	Side of membrane	GOCC	396	1.39E−11
GO:0045335	Phagocytic vesicle	GOCC	46	3.58E−11
GO:0016469	Proton-transporting two-sector ATPase complex	GOCC	35	1.78E−10
GO:0098796	Membrane protein complex	GOCC	803	1.78E−10
GO:0044437	Vacuolar part	GOCC	317	2.82E−10
GO:0005774	Vacuolar membrane	GOCC	303	5.79E−10
GO:0009897	External side of plasma membrane	GOCC	232	5.79E−10
GO:0015078	Hydrogen ion transmembrane transporter activity	GOMF	77	3.46E−09
GO:0004896	Cytokine receptor activity	GOMF	82	1.57E−08
GO:0036442	Hydrogen-exporting ATPase activity	GOMF	24	3.01E−08
GO:0019829	Cation-transporting ATPase activity	GOMF	52	4.24E−07
GO:0035586	Purinergic receptor activity	GOMF	21	6.63E−07
GO:0042625	ATPase coupled ion transmembrane transporter activity	GOMF	53	1.00E−06
GO:0003823	Antigen binding	GOMF	51	1.14E−06
GO:0016820	Hydrolase activity, acting on acid anhydrides, catalyzing transmembrane movement of substances	GOMF	86	1.21E−06
GO:0015077	Monovalent inorganic cation transmembrane transporter activity	GOMF	241	2.68E−06
GO:0001614	Purinergic nucleotide receptor activity	GOMF	17	3.11E−06
GO:0006955	immune response	GOBP	875	1.04E−69
GO:0050776	Regulation of immune response	GOBP	472	1.26E−44
GO:0006952	Defense response	GOBP	1 014	1.28E−44
GO:0002252	Immune effector process	GOBP	566	1.08E−41
GO:0002250	Adaptive immune response	GOBP	275	8.39E−40
GO:0045321	Leukocyte activation	GOBP	630	2.17E−39
GO:0002682	Regulation of immune system process	GOBP	987	4.64E−39
GO:0045087	Innate Immune response	GOBP	430	2.15E−37
GO:0002443	Leukocyte mediated immunity	GOBP	242	1.29E−36
GO:0001775	Cell activation	GOBP	729	4.04E−36

**Table 8:** Cluster identities—characteristic genes

Gene	Fold change	Adj. *P*-value
A. Mesenchymal stem cells		
* Pdgfra*	2.535 350 835	0
* Prrx1*	2.107 476 632	0
B. Satellite cells/stromal progenitors		
* Pax7*	2.874 586 669	0
C. Schwann cells/neural progenitors		
* Plp1*	22.637 784 39	0
* Cnp*	12.449 861 55	0
* Dhh*	2.683 388 559	0
D. Myofibroblasts		
* Acta2 (aSMA)*	24.233 088 11	0
* Mylk*	10.489 084 02	0
E. Endothelial/vascular progenitor cells		
* Cdh5 (VeCadherin)*	11.049 161 63	0
* Pecam1*	10.105 707 91	0
* Cd34*	2.496 854 666	0
F. Lymphocytes		
* Ms4a1*	6.438 651 332	0
G. Myeloid cells		
* Ccr2*	4.634 343 897	0
* Mrc1*	4.064 399 087	0
* Fcgr1*	2.972 696 05	0
* Ccr1*	2.366 385 493	0
* S100a9*	7.099 537 959	0
* Cd209a*	1.899 307 918	1.11E−228

### Mesenchymal progenitor cells, instead of endothelial lineage cells, comprise the majority of cells within traumatic HO lesions

**Fig. 3:**
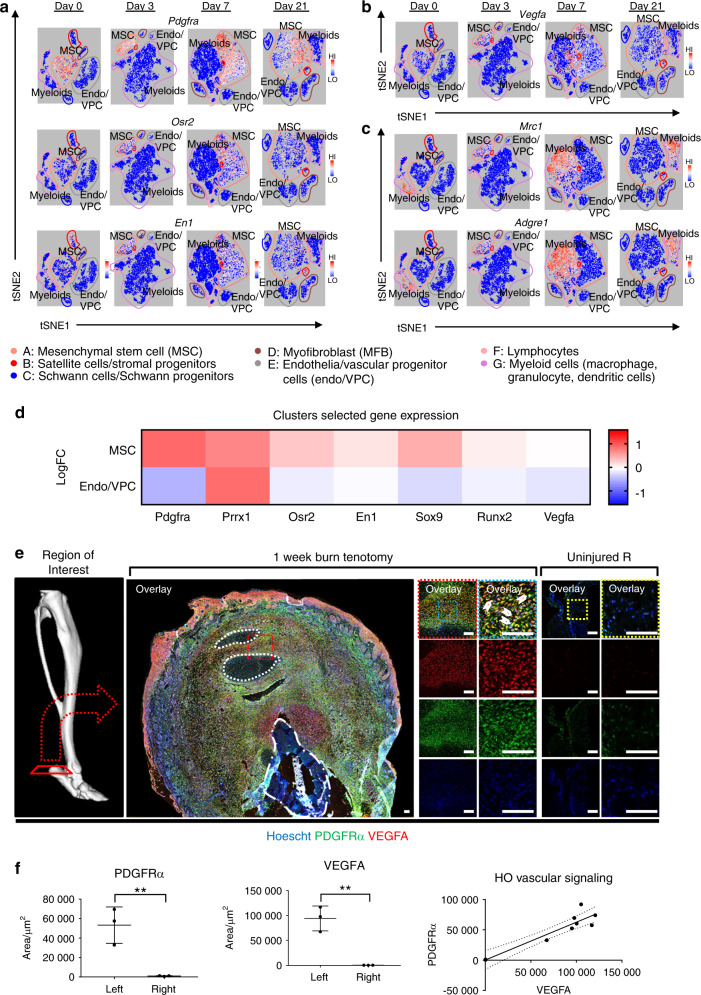
Mesenchymal progenitor cells comprise the majority of VEGFA expressing cells within HO lesions. Clusters from 10× RNA sequencing across days 0, 3, 7, and 21 timepoints. Expression profiles corresponding to **a** mesenchymal progenitor cells: *Pdgfra, Osr2, and En1*; **b** *Vegfa*; **c** and macrophages: *Mrc1* and *Adgre1*. **d** Expression heatmap delineating timecourse for log-fold changes in expression for genes of interest within (Top) mesenchymal stem cell cluster A and (Bottom) endothelial cells/vascular progenitor cells cluster E. Red and blue signify upregulation and downregulation respectively. **e** Representative immunolabeling from burn/tenotomy on wild-type mice 1 week after injury. *n* = 3/group. Color dotted squares designate magnified regions. White dotted lines circumscribe residual bellies of Achilles tendon. Dash dot lines outline reference native bone (calcaneus). Scalebars represent 100 μm. **f** Quantifications for relative signal in 1-week injury site (left leg) vs. uninjured contralateral limb (right leg) following automated thresholding (Yen). Linear regression of PDGFRα vs. VEGFA demonstrates significant correlation

Unsupervised clustering yielded seven unique clusters (Fig. 2c), representing clusters of infiltrating inflammatory cells including macrophages, dendritic cells, and granulocytes, lymphocytes, endothelial cells/vascular progenitors, satellite cells with putative stromal progenitors, and progenitor subpopulations including mesenchymal stem cells, stromal progenitors, neural progenitors, and vascular progenitors. The mesenchymal stem cell population (cluster A) exhibited a remarkably constrained expression of *Pdgfra, Osr2, and En1* (Fig. 3a), all markers for a variety of progenitor populations reported in existing literature. *Vegfa* expression was indeed found in the *Pdgfra* expressing population across all timepoints (Fig. 3b). Interestingly, *Vegfa* expression was also found in macrophage populations after injury (Fig. 3c). Visualized as a timecourse, the mesenchymal stem cell cluster seems to become more heterogenous as a function of time, differentiating into a population with diluted enrichment in *Pdgfra, Prrx1*, and *Vegfa*, along with the expected expression of chondrogenic (*Sox9*) and preosteoblast (*Runx2*) markers (Fig. 3d, top). This expression profile is starkly contrasted by robust downregulation in chondrogenic and osteoblastic genes in the vascular cluster (Fig. 3d, bottom), further corroborating that these cells are not the progenitors for HO formation, nor do they produce VEGFA. Simultaneously referencing the expression profiles (Fig. 3a, b), *Vegfa* seems to correspond strongly with a subpopulation of the mesenchymal stem cell cluster strongly expressing *Pdgfra*. Immunofluorescent histology confirmed robust synthesis of VEGFA at the injury site, colocalizing with PDGFRα positive cells (Fig. 3e), with significant increases in both signals compared with contralateral control and a direct correlation between PDGFRα and VEGFA signal (Fig. 3f).

### *Vegfa* is variably expressed across MSC progenitors and their differentiated progeny

**Fig. 4:**
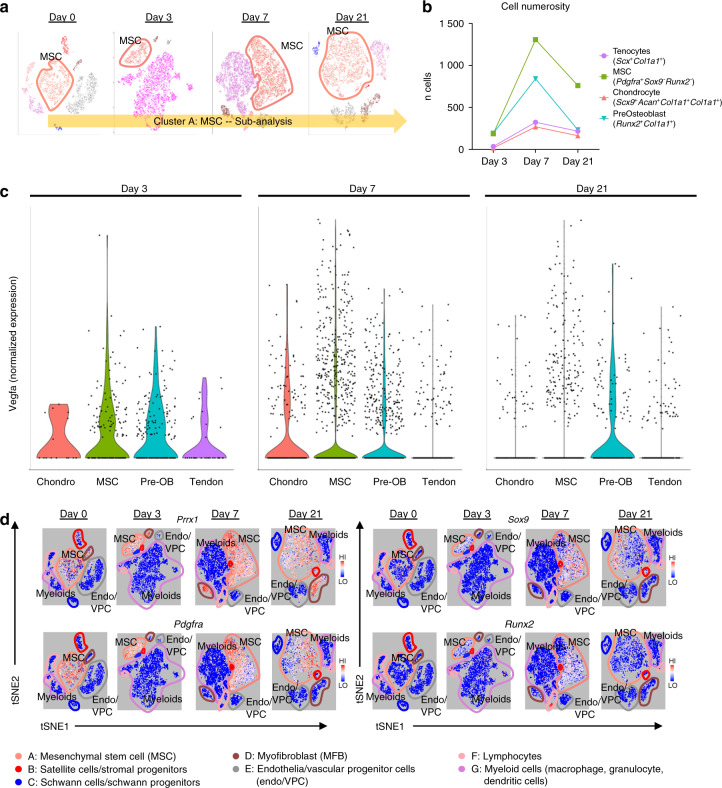
*Vegfa* is variably expressed amongst MSC progenitors and their differentiated progeny. **a** Schematic of cluster subanalysis of MSC cells from single-cell RNA sequencing. **b** Discrete counts of cell types and classification criteria. **c** Expression levels of *Vegfa* across all indicated cell types. **d** Expression profiles of characteristic markers *Prrx1*; mesenchymal progenitors: *Pdgfra;* chondrocytes: *Sox9;* and preosteoblasts: *Runx2*

In order to characterize the MSC cluster with more granularity, we performed a subanalysis on the interesting MSC cluster (Fig. 4a) in light of the suggestive expression profiles pointing to differentiating/differentiated cells like chondrocytes (*Sox9*) and preosteoblasts (*Runx2*) (Fig. 3d). Transcripts profiling tenocytes (*Scx*^*+*^*Col1a1*^*+*^), MSC (*Pdgfra*^*+*^*Sox9*^*-*^*Runx2*^*-*^), chondrocytes (*Sox9*^*+*^*Acan*^*+*^*Col1a1*^*+*^*Col3a1*^*+*^), and preosteoblasts (*Runx2*^*+*^*Col1a1*^*+*^) were used to stratify subgroups within the MSC cluster ‘A.’ These cells were observed to peak In number at day 7, with MSCs (day 7: 1 309 MSCs vs. 269 chondrocytes, 842 preosteoblasts, and 324 tenocytes) the dominant subpopulation comprising this MSC cluster and were subsequently analyzed for their level of *Vegfa* expression as shown in resulting violin plots (Fig. 4c). *Vegfa* was found in low levels within residual tendon that was attenuated by day 7. Conversely, at day 3, MSCs along with chondrocytes, and preosteoblasts to a lesser degree were found to be enriched in *Vegfa*. This transiently dissipated for both MSCs and chondroblasts by day 7. However, more terminally differentiating cells, pre-osteoblasts were found to propagate this *Vegfa* signal even into day 21. With this sustained signaling from MSCs through more differentiated progeny, we investigated a suitable candidate driver that could simultaneously attenuate *Vegfa* expression across the predominant MSCs, but also the spectrum of their differentiation following musculoskeletal polytrauma. We observed *Prrx1* to overlap robustly with not only *Pdgfra* cells, but also *Sox9* and *Runx2* subpopulations as well (Fig. 4d). Given confirmation of transcriptional and translational expression of *Vegfa* from mesenchymal stem cells and their progeny in the injury niche, we hypothesized that conditional knockout in these cells would produce a reduction in posttraumatic HO.

### Conditional genetic deletion of VEGFA from Prx1 lineage cells decreases posttraumatic extremity HO

**Fig. 5:**
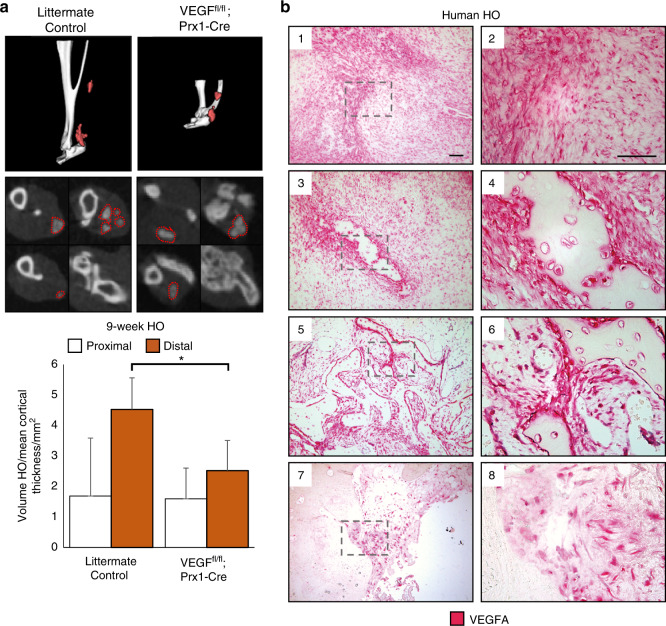
VEGFA knockout and molecular inhibition attenuates formation of HO in the distal hindlimb. Following 9 weeks burn tenotomy, **a** *Prx*-Cre;*Vegf*^*fl/fl*^; treated WT mice exhibit lower volumes of heterotopic ossification than their corresponding controls at 800 HU. Prx-Cre HO volumes normalized by mean cortical thickness (mm) due to congenital phenotype. *n* = 4–9/group. Bars represent means and SD. *P < 0.05 vs. Control by Student’s *t* test. **b** Magnified images of human HO samples immunolabeled for VEGFA: (1,2) pre-HO stroma, (3,4) early HO formation with surrounding stromal fibroblasts, (5,6) intermediate HO, and (7,8) mature HO with terminal ossification. VEGFA indicated by red, scalebar indicates 100 μm

Having identified an increase in vascularity at a traumatic HO site and characterized a potential source for vascular signal, we next set out to assess the role of *Prx1*+ lineage cell derived VEGFA. Our single-cell RNA sequencing data reaffirm co-expression of *Pdgfra* and *Prrx1* (Fig. 4d), corroborating our previously demonstrated work showing cells of the *Prx1*+ lineage define the HO progenitor cells.^34^ In addition, VEGFA has been shown to be produced by osteoprogenitor cells and to drive bone development.^50^ Recent studies also demonstrate that VEGFA from cells marked by Col2CreER-mediated recombination, but not vascular cell lineage (Flt1) functions as a survival and differentiation factor for chondrocytes.^51^ Thus, to evaluate the role of VEGFA on ectopic cartilage formation, we deleted VEGFA from cells of the *Prx1+* lineage. Following VEGF deletion from *Prx1+* MSCs via *Prx1*-cre/*Vegf*^*fl/fl*^, these mutant mice produced significantly less HO in the distal hindlimb (44% relative reduction, *P* = 0.036 after normalization by cortical thickness due to stunted limb phenotype) when induced by burn/tenotomy (Fig. 5a). Given the density of PDGFRα^+^ cells observed with histology, the reduction in ectopic bone formation following selective gene knockout, implicates VEGFA expression and production by MSCs as a major niche in the acute inflammatory response after burn/tenotomy. Additional histological analyses of injury sites of *Prx1*-cre/*Vegf*^*fl/fl*^ 9 weeks post injury exhibit a profound absence i.e. significant ablation of VEGFA signaling in the deletion mice compared with littermate controls, despite comparable levels of PDGFRα^+^ cells (Supplemental Fig. 2) validating the degree of gene ablation.

Finally, histology was obtained from human HO specimens with diverse morphologies that reflect the full histologic spectrum of human disease (Fig. 5b, *n* = 10 human specimens). VEGFA immunostaining was performed, which paralleled our findings from mouse tissues. In early spindle cell lesions before frank ossification (Fig. 5b1,2), a high density of VEGF immunoreactive cells were present. In other areas with early and inconscipuous amounts of woven bone, noticeably stronger VEGF immunoreactivity was present within bone-lining cells (Fig. 5b3,4). These findings were consistent across specimens and in areas of more conspicuous lamellar bone (Fig. 5b5,6), in which bone-lining cells were the again prominently VEGF immunoreactive. In samples with HO of a thicker, more ‘cortical’ appearance (Fig. 5b7,8) bone-lining cells continued to show VEGF immunoreactivity while osteocytes within thicker bone fragments showed little staining. Thus, and across human samples, VEGF highlights similar populations of HO stromal progenitor cells and osteoblasts as in our mouse models, suggesting a potentially similar pathophysiology and disease course.

## Discussion

HO is the formation of ectopic bone highly reliant on angiogenesis to progress through endochondral ossification to mature into its mineralized, final form. VEGFA is regulated by many transcription factors including HIF1a which is thought to be a major stimulus in cancer, bone development, and bone pathology.^19,34,50,52^ Having identified the central role of HIF1a in traumatic HO, we set out to determine whether VEGFA, a downstream signaling mediator of HIF1a, is involved in HO formation.

Previous studies have shown that VEGFA orchestrates ossification during normal bone development.^53^ Furthermore, elegant studies have demonstrated that VEGFA produced by cells of Osx-Cre lineage regulate blood vessel recruitment and early osteoblast differentiation^21^ and that VEGFA produced by *Col2*-Cre-marked cells is necessary for chondrocyte survival.^51^ We chose to target VEGFA in cells of the Prx1-Cre lineage as we were the first to identify these cells as the HO progenitor cells,^34^ a decision further supported by our transcriptomic and histologic data correlating *Prrx1* and *Pdgfra* expression. Notably, in our model, chondrocytes provided limited contribution to *Vegfa* expression levels. However, preosteoblasts were found to be the responsible cell population for *Vegfa* expression at later timepoints (D21), demonstrating that in addition to recruited MSCs, their differentiating progeny propagated the robust expression *Vegfa*. While MSCs were the dominant source for *Vegfa* expression both in magnitude and volume (Fig. 4), the heterogeneity of *Vegfa* also deriving from chondroblasts and preosteoblasts further reinforced our strategy in producing VEGFA knockout via a Prx1 driver. Of particular note from a bioinformatics perspective, while the overwhelming majority of clusters demonstrated high specificity in alignment, two clusters A and D showed moderate levels of correspondence to each other following the initial pseudo clustering (Fig. 2a). These clusters correspond to related progenitor cells, both expressing a common ancestry given their prrx1 enrichment. However, the musculoskeletal mesenchymal progenitor population (D) exhibited sparse VEGFA signaling, essentially rendering this an inert subpopulation. Here we show that genetic loss of VEGFA from the mesenchymal stem cell population (along with some chondrocyte and preosteoblasts) is sufficient to significantly reduce HO volume. VEGFA inhibition in cells of the Prx-Cre lineage did not, however, completely mitigate all HO formation likely due to alternative sources for VEGFA as demonstrated in our single-cell RNA sequencing, namely inflammatory cells.^51,54,55^ Despite previous literature implicating vascular sources of VEGFA secreted in a cell autonomous manner,^48^ our sequencing data concretely demonstrated a notable absence of *Vegfa* transcripts. In addition, these cells did not express common markers of chondrogenic and osteoblastic differentiation, cementing their supportive role in the periphery of the developing ectopic bone.

Histological analysis of our acute injury model demonstrated a lack of spatial proximity between *Cdh5*-creERT2 cells and VEGFA within the HO region when activated just prior to injury. Cells positive for *Cdh5*, however, were present in a large amount surrounding the mesenchymal condensation that underwent endochondral ossification corroborating our sequencing results and the *Cdh5*+ cell’s important supportive role to the HO niche. This presence of *Cdh5+* cells also corresponded with an increase in blood flow as well as blood vessels likely derived from the identified EPCs, which is consistent with the rubor and swelling seen at a joint predisposed to HO prior to radiographic evidence.

Two pathways could explain our observed phenotype of HO inhibition following loss of mesenchymal-derived VEGFA: decreased angiogenesis secondary to loss of the trophic signal leading to an indirect deleterious effect on endochondral ossification or direct phenotypic changes to the mesenchymal cells themselves. Interestingly, our mesenchymal stem cell population did not exhibit upregulation of any VEGF receptors, including *Flt1*/VEGFR1, *Kdr*/VEGFR2, or *Flt4*/VEGFR3 (data not shown) rendering them unresponsive to VEGFA. This is largely concordant with previous literature characterizing VEGFA activity in fetal tissues.^23^ Given the large influx of inflammatory cells including VEGFA expressing macrophages, one could presume that there should be enough residual VEGFA at the locus of injury to rescue the loss of mesenchymal-derived VEGFA, especially with the former pathway. Given our observed reduction in HO, it seems the latter pathway is more likely. Indeed, previous work by Zelzer et al. ^23^ demonstrated that VEGFA loss from mesenchymal stem cells leads to aberrant growth plate development despite no changes to surrounding vasculature, instead pointing to transcriptional changes within the differentiated mesenchymal cells and chondroblasts themselves. Furthermore, in this paradigm, the partial bone phenotype is unsurprising as *Pdgfra/Prrx1* cells highly expressing VEGFA were a subpopulation of a larger pool of progenitors. Nevertheless, the targeted ablation of VEGFA was observed to produce marked, clinically-relevant reductions of posttraumatic HO. As such, future work could involve utilization of selective, deliverable next-generational inhibitors such as siRNAs for cell-specific knockdowns to attenuate HO formation.

This work is also the first to our knowledge to further characterize the role for novel progenitor-cell populations rapidly expanding in the literature. Subpopulations characterized as dermal lineage progenitors like engrailed1 (*En1*)^56^ and fibro-adipogenic progenitors like odd-skipped related-1/2 (*Osr1/2*)^57^ were found in the larger *Pdgfra* cluster, implicating *En1* and *Osr1/2* expressing cells as subsets of a larger progenitor population involved with HO formation. These cells appear transcriptionally distinct from *Vegfa* expressing progenitors, suggesting next steps that will be necessary to further characterize the differentiation capacity of these cells in the HO niche and to what degree they differ in the disease course. In addition, given previous work demonstrating the spatial variation of different lineages i.e. concentration of Prx-positive cells in the distal hindlimb, these separate cells with progenitor phenotypes may perhaps mark more proximal contributing cells to HO formation. Downstream high-throughput analyses facilitated by Next-Gen sequencing modalities as performed in this work will offer additional insights into important phenotypes and candidate targets.

This study has several notable limitations. Our RNA-sequencing results did not offer substantial resolution between mature endothelium (Supplemental Fig. 1) and EPCs. However, the flow cytometry did mark this separation well. Furthermore, this data would suggest that analysis of VeCadherin endogenous reporters would be likely indicating both mature endothelium in addition to the EPCs on histology. Thus, a complete absence of signal necessarily still validates an absence of EPCs. We chose to use *Prx*-Cre which will mark all cells from embryonic and postnatal development rather than an inducible Cre given our previous studies that demonstrate their central role in HO using this model.^34^ In addition, we do not delineate whether VEGF stimulates MSC differentiation^58^ or just promotes chondrocyte survival. In addition, because of our approach to characterize perturbations of cellular populations found at baseline, these constraints limited are ability to characterize the cell populations with further granularity i.e. myeloid cells vs. individual clusters for macrophages, granulocytes, and dendritic cells. This leaves us potentially susceptible to artifacts in results specifically due to dilution of clusters (as seen in the *Vegfa* expression patterns in the mesenchymal stem cells.

Notwithstanding these limitations, our findings demonstrate that mesenchymal-derived VEGFA is a crucially expressed signal in the genesis of HO, suggesting that therapies targeting VEGFA may present a reasonable option for reducing this debilitating disease and expanding the repertoire for its prevention. Subsequent studies are required to determine the efficacy and safety profile of targeted siRNA delivery systems and existing systemic anti-VEGFA antibodies such as bevacizumab in more directed wound healing models for HO patients.

## Materials and methods

### Ethics statement

All animal experiments described were approved by the University Committee on Use and Care of Animals at the University of Michigan, Ann Arbor (Protocols: #05909 and 07930). This study was carried out in strict accordance with the recommendations in the *Guide for the Care and Use of Laboratory Animals of the National Institutes of Health*.^59^ All animal procedures were carried out in accordance with the guidelines provided in the *Guide for the Use and Care of Laboratory Animals: Eighth Edition* from the Institute for Laboratory Animal Research.^60^

### Animals

Mice evaluated for ectopic bone were wild-type C57BL/6J (Jackson Laboratories), Cdh5-Cre/tdTomato^fl/fl^ ^61^, *VeCadherin(Cdh5)-creERT2/tdTomato*^*fl/fl*^ ^62^ (a gift from Dr Luisa Iruela Arispe, UCLA), *Prx1-cre*^34^*/Vegfa*^*fl/fl*^, or *Prx1-cre/Vegfa*^*fl/fl*^ littermate controls. All breeding was performed at the University of Michigan in facilities managed by the Unit for Laboratory Animal Medicine. CreERT reporters were induced with two doses of 1 mg tamoxifen in mice one week prior to burn/tenotomy for adequate washout by the time of injury.

### Injury models

All mice received presurgical analgesia consisting of 0.06 mg·kg^−1^ buprenorphine for 48 h, followed by anesthesia with inhaled isoflurane, and close postoperative monitoring with analgesic administration.

Mice received 30% total body surface area partial-thickness burn on a shaved dorsum followed by transection of the left Achilles tendon. Dorsal burn was induced using a metal block heated to 60 °C in a water bath and applied to the dorsum for 18 s continuously. HO anlagen was observed by week 3 with mature bone formation visible by microCT by 9 weeks.

### In vivo imaging system

Mice were anesthetized via inhaled isoflurane and depilated with Nair. Specimens were imaged before injection for baseline fluorescence levels at 770-nm wavelength using an IVIS Spectrum system (PerkinElmer 124262). Per manufacturer’s instructions, 100 μL of 20 μmol·L^−1^ AngioSense 750EX (PerkinElmer NEV10011EX) solution was injected into the tail vein followed by re-imaging 24 h post injection at excitation/emission of 750/800 nm. AngioSense protocol was performed 20 h post burn/tenotomy (*n* = 3/group).

### 10× Single-cell RNA sequencing

Baseline uninjured tendon (day 0) and post surgery day 3, 7, and 21 harvested tissue samples were digested for 45 min in 0.3% Type 1 Collagenase and 0.4% Dispase II (Gibco) in Roswell Park Memorial Institute (RPMI) medium at 37 °C under constant agitation at 120 r·min^−1^. Digestions were subsequently quenched with 10% FBS RPMI and filtered through 40 μm sterile strainers. Cells were then washed in PBS with 0.04% BSA, counted and resuspended at a concentration of ~1 000 cells per μL. Cell viability was assessed with Trypan blue exclusion on a Countess II (Thermo Fisher Scientific) automated counter and only samples with >85% viability were processed for further sequencing.

Single-cell 3′ library generation was performed on the 10× Genomics Chromium Controller following the manufacturer’s protocol for the v2 reagent kit (10× Genomics, Pleasanton, CA, USA). Following generation of single-cell gel bead-in-emulsions (GEMs), reverse transcription was performed and the resulting Post GEM-RT product was cleaned up using DynaBeads MyOne Silane beads (ThermoFisher Scientific, Waltham, MA, USA). The cDNA was amplified, SPRIselect (Beckman Coulter, Brea, CA, USA) cleaned and quantified then enzymatically fragmented and size selected using SPRIselect beads to optimize the cDNA amplicon size prior to library construction. An additional round of double-sided SPRI bead cleanup is performed after end repair and A-tailing. Another single-sided cleanup is done after adapter ligation. Indexes were added during PCR amplification and a final double-sided SPRI cleanup was performed. Libraries were quantified by Kapa qPCR for Illumina Adapters (Roche) and size was determined by Agilent tapestation D1000 tapes. Read 1 primer sequence are added to the molecules during GEM incubation. P5, P7 and sample index and read 2 primer sequence are added during library construction via end repair, A-tailing, adapter ligation and PCR. Libraries were generated with unique sample indices for each sample. Libraries were sequenced on a HiSeq 4000, (Illumina, San Diego, CA, USA) using a HiSeq 4000 PE Cluster Kit (PN PE-410-1001) with HiSeq 4000 SBS Kit (100 cycles, PN FC-410-1002) reagents, loaded at 200 pmol·L^−1^ following Illumina’s denaturing and dilution recommendations. The run configuration was 26 × 8 × 98 cycles for Read 1, Index, and Read 2, respectively. Cell Ranger Single Cell Software Suite 1.3 was used to perform sample de-multiplexing, barcode processing, and single-cell gene counting (Alignment, Barcoding and UMI Count) at the University of Michigan Biomedical Core Facilities DNA Sequencing Core.

A total of ~200-500 million reads were generated from the 10× Genomics sequencing analysis for each replicate. The sequencing data were first preprocessed using the 10× Genomics software Cell Ranger (10× Genomics Inc., Pleasanton, CA, USA) and aligned to mm10 genome (deposited to the Gene Expression Omnibus database: GSE126060). Downstream analysis steps were performed using Seurat. Cells with fewer than 500 genes, more than 10% of reads mapping to the mitochondrial genome, or more than 60 000 UMIs; and genes expressed in fewer than 10 cells per replicate, were filtered for quality control. The downstream analysis steps for each sample type include normalization, scaling, dimensionality reduction (PCA, t-SNE, and UMAP), unsupervised clustering, and the discovery of differentially expressed cell-type specific markers. We verified the absence of the within-time-point batch effect by visual inspection of the per-sample contribution in the per-day merged set, once we pooled together samples from each timepoint (Fig. 2b). In order to characterize the induced changes in cellular populations following burn/tenotomy injury, provisional clusters were identified unsupervised clustering followed by centroid rank correlation comparison within and between timepoints (days 0, 3, 7, and 21). Provisional clusters that failed to align across these comparisons were discarded, leaving behind cells and clusters that were identified in the steady state (day 0) timepoint and subsequently perturbed across days 3, 7, and 21. Final clusters were refined by pooling cells from all the timepoints to create a merged set; correcting for batch effect of the merged set; and performing a supplemental unsupervised clustering. Expression profiles of known markers, GO term and KEGG pathway analysis, along with characteristic expression profiles were utilized to identify the cell types of each cluster. FDR adjustments were performed to determine statistical significance of gene expression fold changes within a cluster vs. all other clusters within the merged set. Enrichment was performed with LRPath. The whole Bioinformatics analysis summarized here is detailed in the supplementary material (Figs. S3–15).

### Histology and immunofluorescence

Histologic evaluation was performed at indicated timepoints in wild-type C57BL/6J and tdTomato endogenous reporter mutants following burn/tenotomy. TdTomato and wild-type hindlimbs were fixed at 4 °C in 4% paraformaldehyde or 10% neutral-buffered formalin. Specimens were subsequently decalcified in 14% (m/v) EDTA solution for 4–6 weeks at 4 °C until deformable manually. Hindlimbs were cryo- or paraffin-embedded and 8 μm–10 μm sections were cut and mounted on Superfrost plus slides (Fisher) and stored at −20 °C.

Paraffin sections were consistently selected from similar regions defined by talus as the anatomical landmark, rehydrated, processed for antigen retrieval in citrate buffer for 20 min at 95 °C–100 °C and blocked with 2% serum, 1% BSA, and 0.1% fish skin gelatin. Human sections were separately treated with trypsin enzymatic antigen retrieval solution (Abcam) for 10 min at 37 °C and blocked with 3% hydrogen peroxide (20 min, RT) and 5% goat serum (1 h, RT). Immunostaining of HO anlagen was performed on paraffin sections with the following antibodies: goat anti-mPDGFR-alpha (R&D Systems, AF1062) and rabbit anti-mouse VEGFA (EMD Millipore, ABS82). Secondary antibodies consisted of donkey anti-rabbit or anti-goat Alexafluor-488 or −594 (Invitrogen). Primary and fluorophore conjugated secondary antibodies were diluted 1:50–100 (2–4 μg·mL^−1^) and 1:200, respectively. Nuclear counterstain was performed with Hoechst 33342 (Life Technologies). Immunostaining of human HO was performed on paraffin sections with polyclonal rabbit anti-mouse VEGFA (Santa Cruz, Cat No. sc-152). Slides were subsequently blocked with BLOXALL solution (Vector) and 2.5% normal horse serum, and incubated with second primary antibody, IMPRESS-AP reagent (Vector) with development and counterstain via Alkaline Phosphatase Substrate and hematoxylin (Vector). Appropriate primary antibody negative controls were run simultaneously with each tested sample.

### Microscopy

Cryosections of tdTomato endogenous reporter mice were imaged with a Zeiss LSM 510-META Laser Scanning Confocal Microscope equipped with a helium neon 1 laser (AF594, RFP). Cryosections for immunolabeled samples were performed on a Leica TCS SP8 Laser Scanning Confocal Microscope with tunable laser whose excitation and emission parameters were set to fluorophore manufacturer’s instructions. Antibody labeled paraffin sections were imaged with epifluorescent upright scope (Olympus BX-51) equipped with DAPI and dual-cube 488 nm/TRITC filters attached to an Olympus DP-70 high-resolution digital camera. Each site was imaged in all channels and were overlaid in Olympus DPViewer or Photoshop before examination and quantifications in ImageJ. Images were adjusted linearly only in brightness and contrast identically across comparison groups for clarity where necessary.

For quantification of positively fluorescence, images were automatically thresholded via Yen method, and then measured across 1–3 separate hpf per biologic specimen (ImageJ, *n* = 1–3/specimen, *N* = 3 specimens/treatment group). Images were then analyzed for area via the standard ImageJ measurement panel.

### Flow cytometry

FACS analysis was performed on the injury site after harvesting the soft tissue of the posterior compartment between the muscular attachment of the Achilles tendon and the calcaneal insertion. Correlating soft tissue was also harvested from the contralateral uninjured leg. Tissue was digested for 60 min in 0.3% Type I Collagenase and 0.4% Dispase II (Gibco) in RPMI at 37 °C under constant agitation. Digestions were quenched with RPMI supplemented with 10% FBS and filtered using a 70-μm sterile strainer, transferred to 15 mL conical tubes, and centrifuged at 300 × *g* for 5 min. Tubes were decanted and washed in PBS three times. Digested specimens were blocked for Fc receptors with anti-mouse CD16/32 for 15 min at 4 °C, and subsequently stained with the following antibodies: Tie2-PE (12-5987-83, eBioscience), CD31-APC (551262, BD Pharmingen), CD133-AF488 (11–1331–82, eBioscience), and CD34-eFluor450 (48–0341–82, eBioscience). Following 30 min of incubation at 4 °C, samples were washed, filtered through a 35 μm mesh strainer, incubated with propidium iodide, and run on a FACSAria II (BD Biosciences) Cell Sorter at the University of Michigan Flow Cytometry Core in the Biomedical Science Research Center. For sorting, after appropriate FSC/SSC, singlets, and viable cells were gated, the CD31 and Tie2 double-negative subpopulation was enriched for CD34 positive EPCs. Tie2 and CD31 double positive subpopulations were further refined for CD133 and CD34 double-negative mature endothelium. Event counts were normalized to total viable cells to control for possible differences in the amount of tissue harvested. Data were then analyzed using FlowJo software.

### MICROFIL imaging

In vivo collateral vessel formation was assessed using MICROFIL. At 9 weeks after burn/tenotomy, wild-type mice were euthanized with cannulation of descending thoracic aorta and venting of inferior vena cava. Mice were perfused with MICROFIL (MV120-blue; Flow Tech Inc.) which was allowed to polymerize overnight at 4 °C and subsequently imaged with μCT (*n* = 2/group).

### MicroCT analysis

Mouse hind limbs were imaged with μCT at 9 weeks post injury (Bruker, 35 μm resolution and 357 μA/70 kV beam). Images were reconstructed and ectopic bone volume formation calculated with Microview (GE Healthcare v2.2, Parallax rc16). All scans were analyzed with threshold Hounsfield units (HU) of 800, 1 250, or 1 800 to determine the gross volume of mineralized tissues. Additional analyses delineated ectopic bone proximal to the tibial-fibular confluence from bone distal to this landmark and were defined as proximal and distal HO respectively.

### Statistical analysis

Primary outcome of interest for *a priori* power analysis is volume of mature ectopic bone formation. To detect 50% decrease from 7.5 mm^3^ in untreated mice at significance level of 0.05 and power of 0.80, three mice were required per group. Means and SD were calculated from numerical data, as presented in text, figures, and figure legends. Bar graphs represent means with error bars specifying one SD. Sample groups were confirmed to be homoscedastic via Levene’s test. Pairwise comparisons were conducted with Student’s *t* test after confirmation for homoscedasticity via *F*-test. *P* values are included in figure legends.

## Supplementary information

Supplemental figures
