# Supplementary material for: Mesenchymal VEGFA induces aberrant differentiation in heterotopic ossification
Source: Bone Res. 2019 Dec 10;7:36. doi: 10.1038/s41413-019-0075-6 (PMC6904752; doi:10.1038/s41413-019-0075-6)
Supplement: Supplementary file 1 — Supplemental figures [file 41413_2019_75_MOESM1_ESM.pdf]

Supplementary material for “Loss of mesenchymal progenitor-derived VEGFA impairs aberrant differentiation in heterotopic ossification”, Hwang. et al

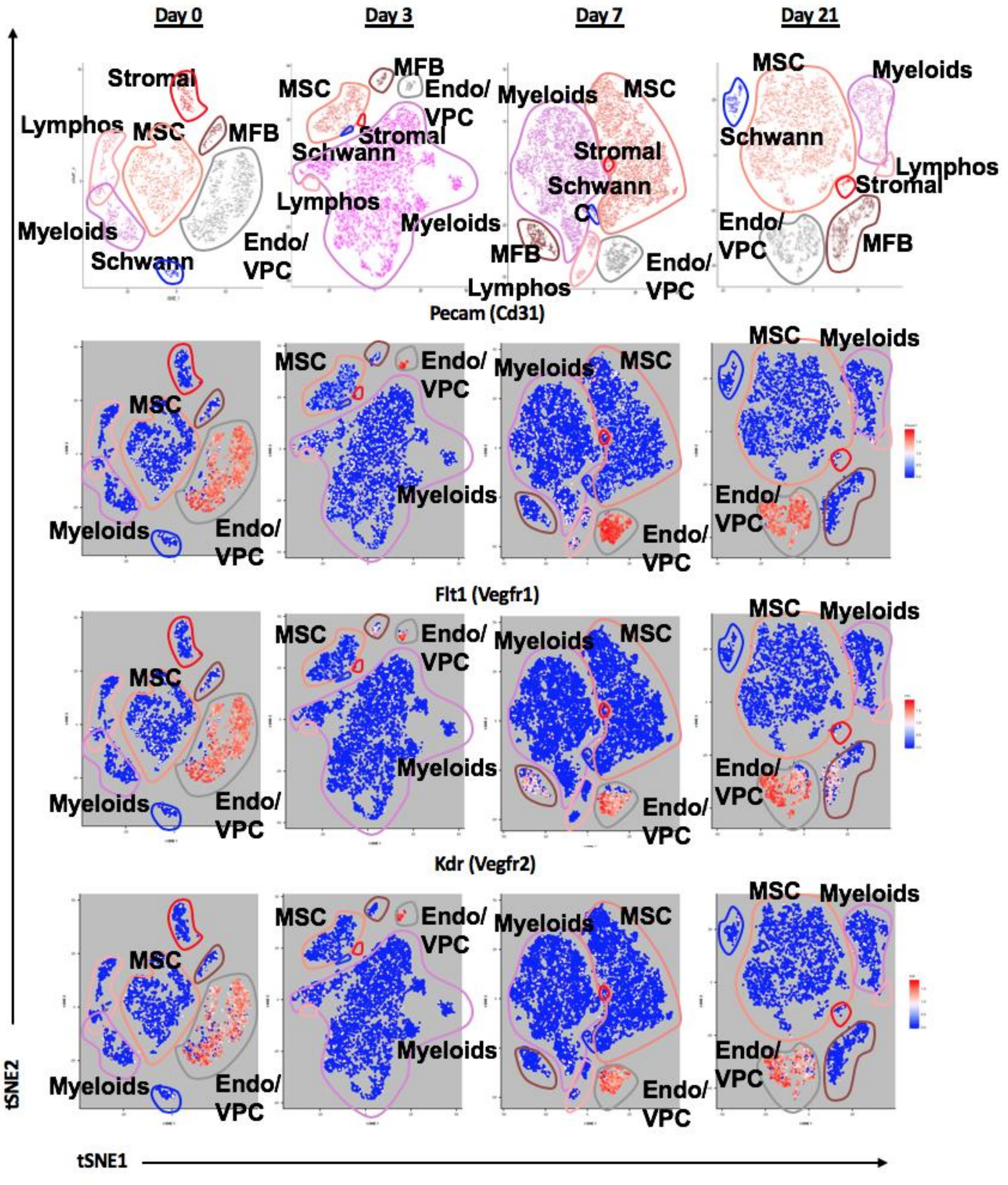

Supplemental Figure 1. **Endothelial/Progenitor cells cluster together in the HO model.** Single cell RNAsequencing demonstrates robust signaling for mature endothelium (*Pecam/Cd31, Flt1/Vegfra, and Kdr/Vegfr2*) within the endothelial/vascular progenitor cell [EPC] clusters across all timepoints.

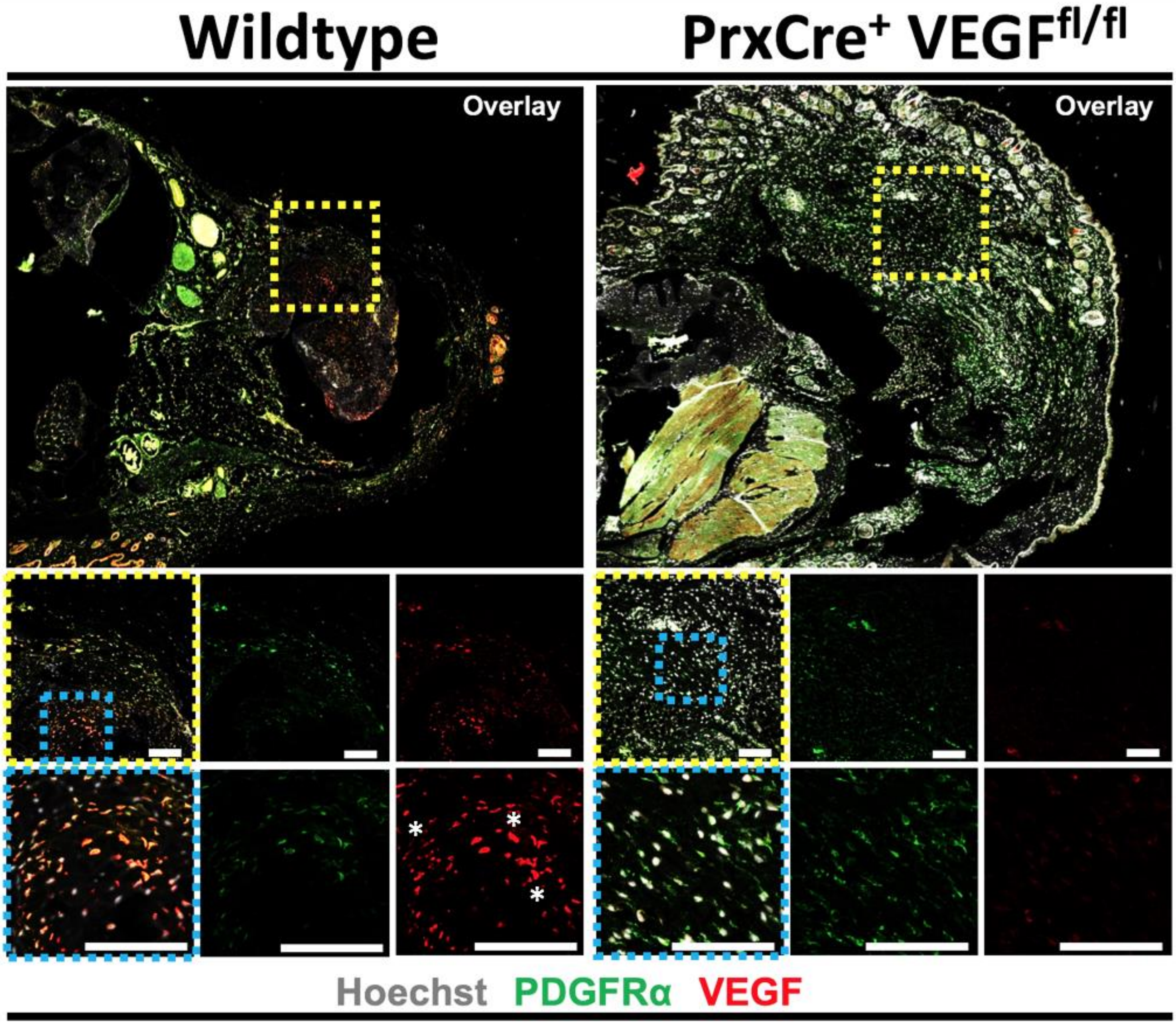

Supplemental Figure 2. Selective VEGFA knockout in Prx-positive mesenchymal stem cells demonstrate significant reduction in VEGF signaling. Immunolabeling for PDGFRα vs. VEGFA demonstrates a significant attenuation of VEGFA signal (white asterisks) in the mutants harboring selective knockout within PrxCre-positive mesenchymal stem cells, despite similar prevalence of mesenchymal stem cells (n=1-2/group).

Bioinformatics analysis (supp. figs 3-15)

The 15 data sets (4 replicates for each of Day 00, Day 07, and Day 21; 3 replicates for Day 03) were analyzed individually and independently. The Seurat 2.3 pipeline [1] was utilized for the analysis.

Data preprocessing

Each set was assigned to a separated Seurat Object (function CreateSeuratObject), with the filters min.cells = 10, and min.genes = 500. The function FilterCells was applied with high thresholds for fraction of the mitochondrial expressed genes and total UMIs of 0.1 and 60,000, respectively. Finally, data were normalized and scaled with the functions NormalizeData and ScaleData (default parameters). By intersecting the genes of all the individual sets we obtained a list of 10572 genes, which will refer to as the common gene set.

**Day**	**Replicate**	**Cells**	**(init.)**	**Genes**	**(init.)**
00	1	855	(1055)	12247	(16423)
00	2	540	(614)	11306	(15824)
00	3	1056	(1194)	11857	(16100)
00	4	839	(952)	11911	(16144)
**Total**		3290		12595	

**Day**	**Replicate**	**Cells**	**(init.)**	**Genes**	**(init.)**
03	1	1915	(2015)	13867	(17826)
03	2	2101	(2186)	14276	(18370)
03	3	1661	(1803)	13625	(17808)
**Total**		5667		14524	

**Day**	**Replicate**	**Cells**	**(init.)**	**Genes**	**(init.)**
07	1	3180	(3405)	14852	(18965)
07	2	3507	(3694)	14810	(18754)
07	3	3595	(3786)	14861	(18898)
07	4	2849	(2964)	14757	(18715)
**Total**		13131		15471	

**Day**	**Replicate**	**Cells**	**(init.)**	**Genes**	**(init.)**
21	1	1510	(1607)	13965	(17995)
21	2	891	(932)	13304	(17468)
21	3	917	(967)	13319	(17477)
21	4	1908	(1986)	14347	(18254)
**Total**		5226		14556	

PCA and t-SNE projections

PCA and t-SNE plots were calculated with Seurat functions RunPCA (considering all the genes of each preprocessed set independently), and RunTSNE (dims.use = 1:10, and do.fast = TRUE) respectively.

From provisional clusters to clusters

Provisional clusters were obtained with unsupervised clustering (Seurat, Louvain algorithm, reduction.type = "tsne", dims.use = 1:2, resolution = 0.1, k.param = 10). This procedure initially produced 13, 9, 13, and 12 provisional clusters for replicate 1-4 of Day 00 respectively; 14, 16, and 13 provisional clusters for replicate 1-3 of Day 03 respectively; 20, 23, 21, and 18 provisional clusters for replicate 1-4 of Day 07 respectively; and 17, 10, 11, and 15 provisional clusters for replicate 1-4 of Day 21 respectively.

Provisional cluster centroids for each replicate of each of the four time points were calculated the average expression over the common gene set. Firstly, provisional clusters were aligned and manually ordered within each set to find similar ones. Secondly, provisional clusters were aligned within the same time point, to find similarities shared by groups of provisional clusters from each replicate of the same time point. Thirdly, provisional clusters from Days 03, 07, and 21 were aligned against the ones of Day 00, to find inter-time point similarities. Some original provisional clusters were further explored by splitting them into two sub-provisional clusters; this allowed the discovery of a better matching community structure, i.e., sharper correspondences in the centroid correlation heat maps. Specifically, one cluster was split in Day 03 replicate 3, and two clusters were split in Day 21 replicate 2-3. Finally, small provisional clusters that failed to align (i.e., by showing a consistently low correlation to all the other provisional cluster, both in the infra- and inter-time points) were discarded; but provisional clusters that showed intra- or inter-time point consistency were kept. Specifically, two provisional clusters from Day 00, replicate 4 (28 and 15 cells); two from Day 03, replicate 2-3 (54 and 24 cells respectively); and two from Day 21 replicate 2-3 (30 and 11 cells, respectively) were considered unaligned and removed from the analysis.

Consolidation

Provisional clusters that showed high heatmap correlation within the same set were merged. The seven provisional clusters of Day 00 were found to be present in all the four sets, and were named A-G (Supplementary Figure 3). For the other time points, provisional clusters were firstly aggregated by considering the internal correlation of each separate set; then, each cluster was aligned against the seven clusters of Day 00 to be named (Supplementary Figures 4-9). Centroid alignment could not always find a clear cluster separation corresponding to A-G of Day 00 for the other time points (e.g., cluster FG and AB in Day 03). To resolve this, we pooled together the cells selected from the previous steps and merged them into an all-time-point set. We normalized and extracted the variable genes (Seurat, FindVariableGenes, y.cutoff = 0.25). We used provisional clusters A/AB, D, and F as anchors to correct for the batch after we observed them consistently translated across the time points in the PCA projection (Supplementary Figure 10). To correct for the batch effect we firstly calculated the centroids for A/AB, D, and E clusters in each time point over the normalized genes. Secondly, we calculated the mean M_t of A, D, and E centroids for each time point $t \in$ (Days 00, 03, 07, 21). Using Day

00 as the reference, we subtracted M_{D00} from the other time points to obtain a Δ_t for Days 03, 07, 21. Finally, we subtracted each Δ_t from the normalized gene expression of all the cells belonging to that time point. PCA calculation after batch correction showed aligned centroids (Supplementary Figure 11.)

The final cluster attribution was obtained by unsupervised clustering (Seurat, Louvain algorithm, reduction.type = "pca", dims.use = 1:10, resolution = 0.025, k.param = 10) applied to the all-time-point set, leading to six clusters (Supplementary Figure 12). Clusters B and C where attributed to a single cluster, but the provisional cluster centroid alignment indicated that B and C should be separated. Therefore, after isolating the BC cluster, we proceeded subclustering BC. Unsupervised clustering (k-means, "Hartigan-Wong", based on the first 10 PCs, k=2) confirmed the cluster labels B and C to be distinct (Supplementary figure 13). The final cluster are presented in Supplementary Figure 14. Provisional and final cluster concordance is reported in Supplementary Table 1.

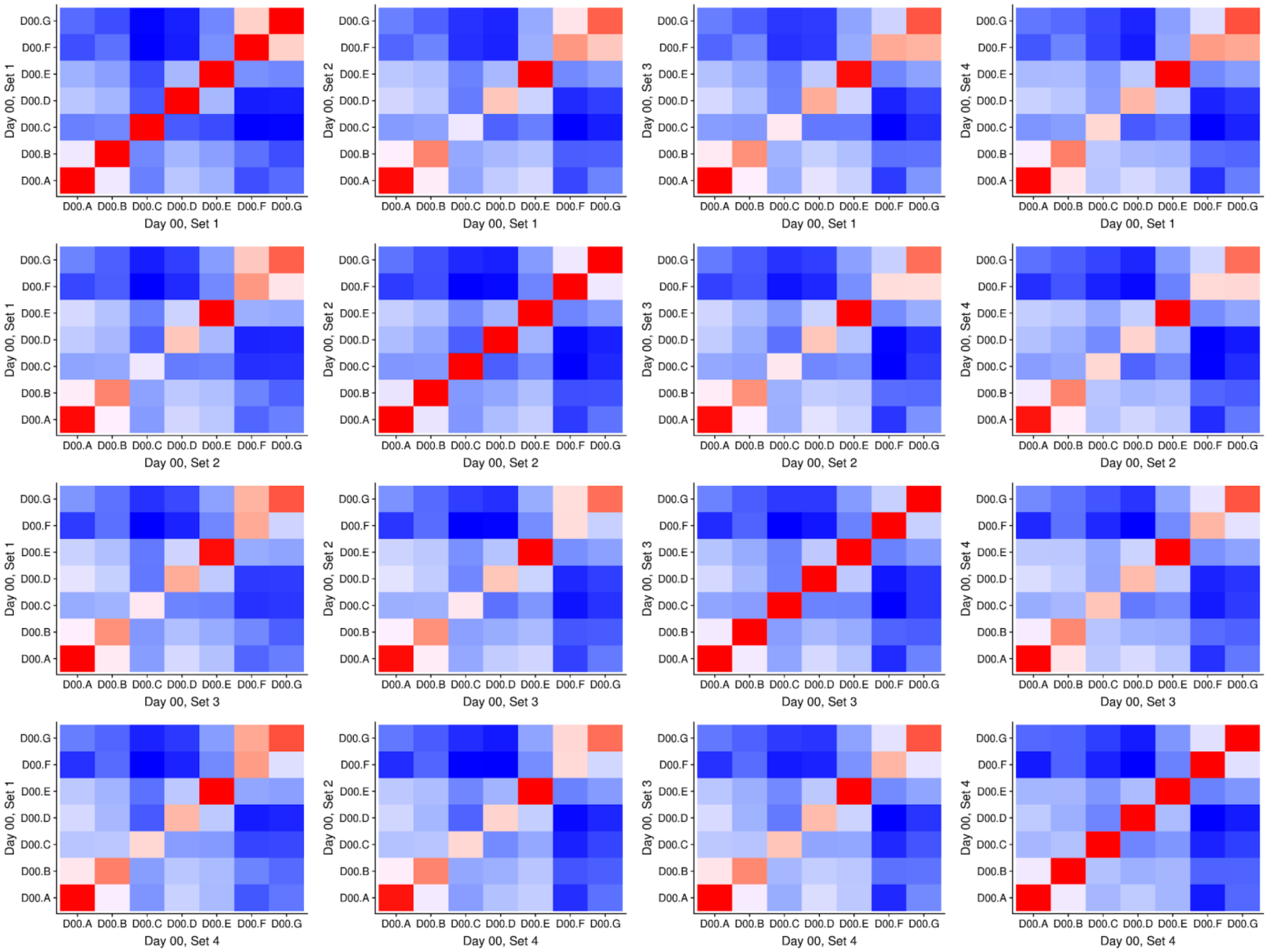

Supplemental Figure 3. Intra-day centroid correlation for the four replicas of Day 00. Clusters correspond well across replicates of each timepoint consistent with no batch effect artifacts.

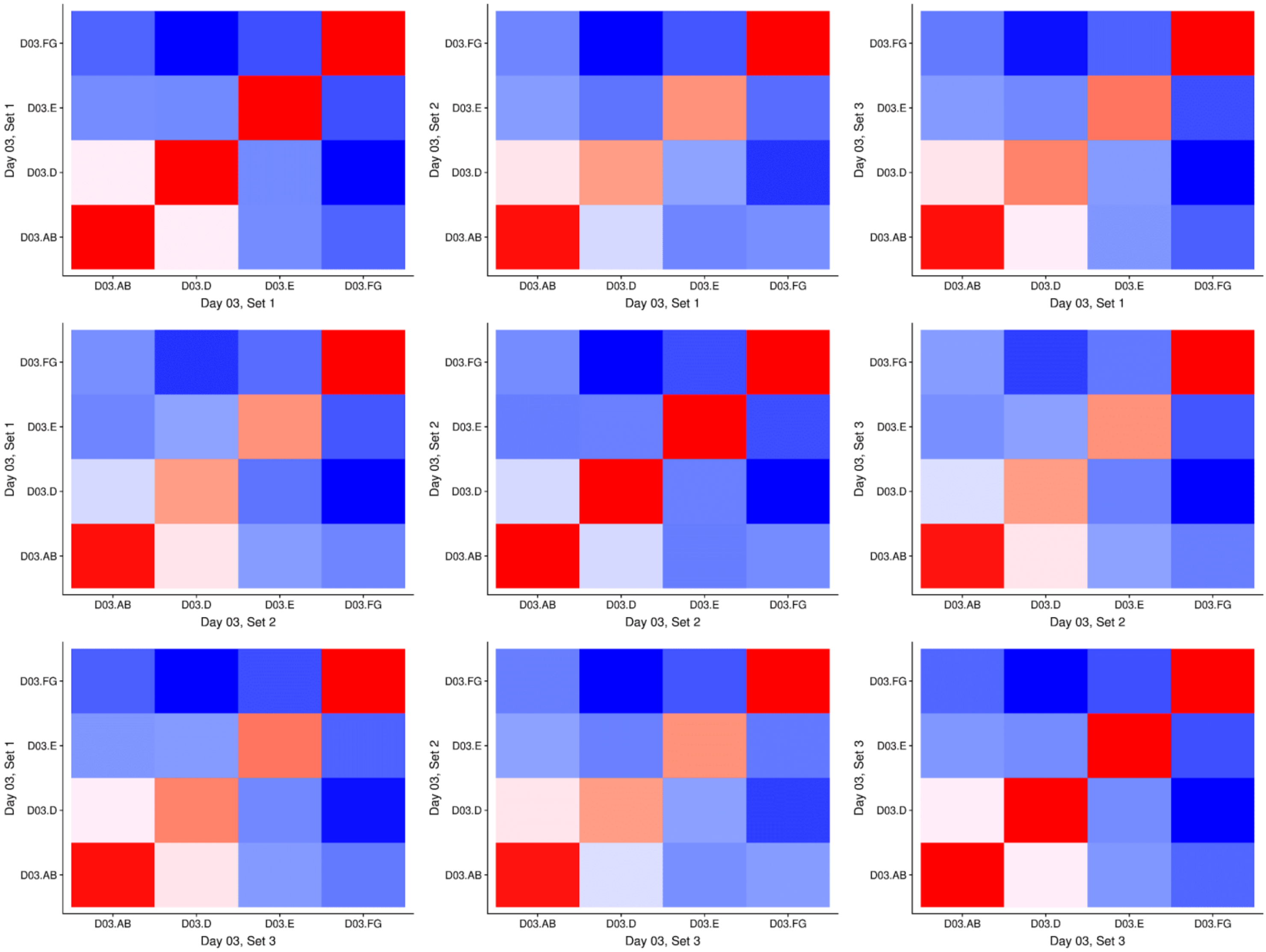

Supplemental Figure 4. Intra-day centroid correlation for the four replicas of Day 03. Clusters correspond well across replicates of each timepoint consistent with no batch effect artifacts.

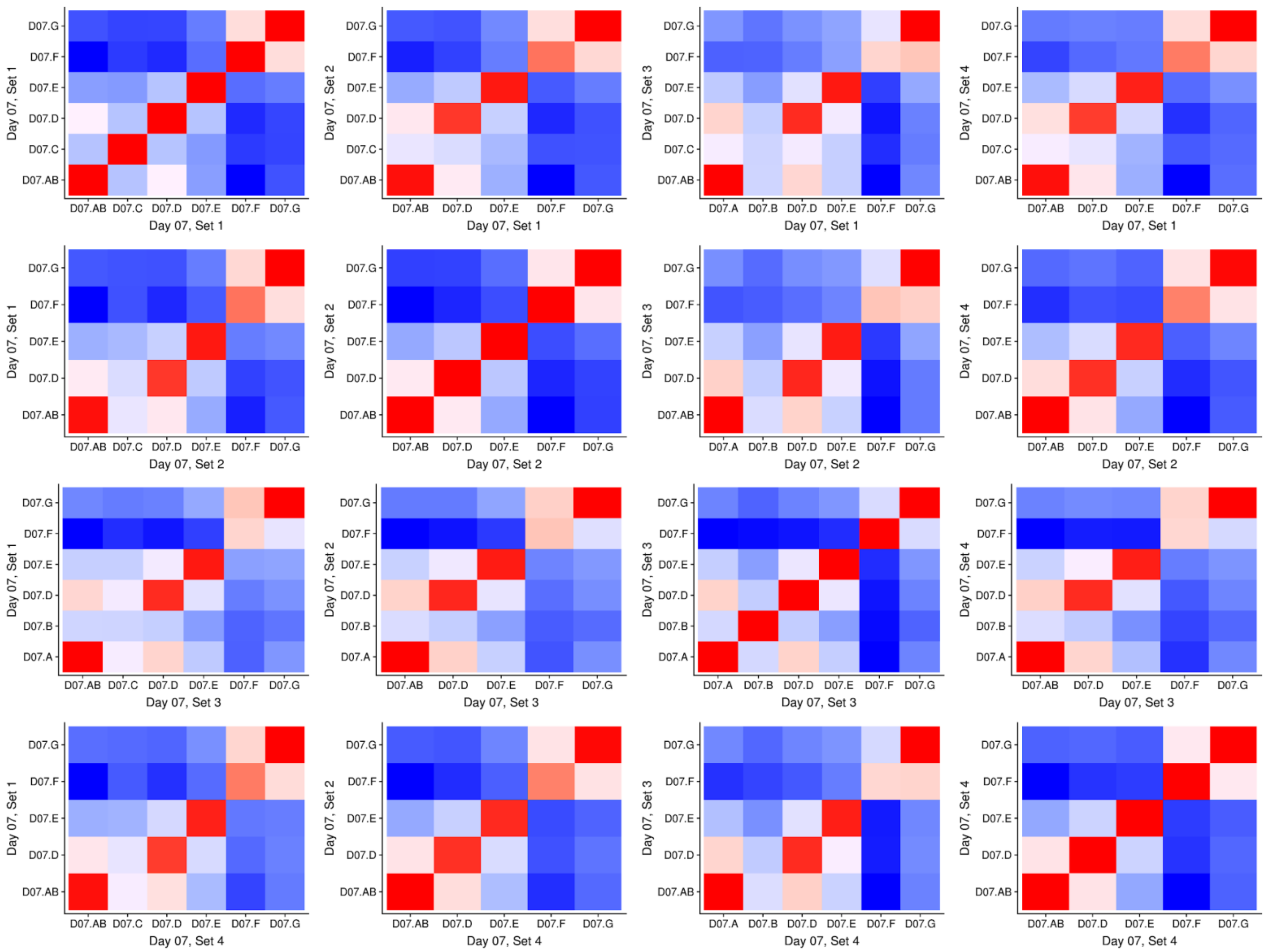

Supplemental Figure 5. Intra-day centroid correlation for the four replicas of Day 07. Clusters correspond well across replicates of each timepoint consistent with no batch effect artifacts.

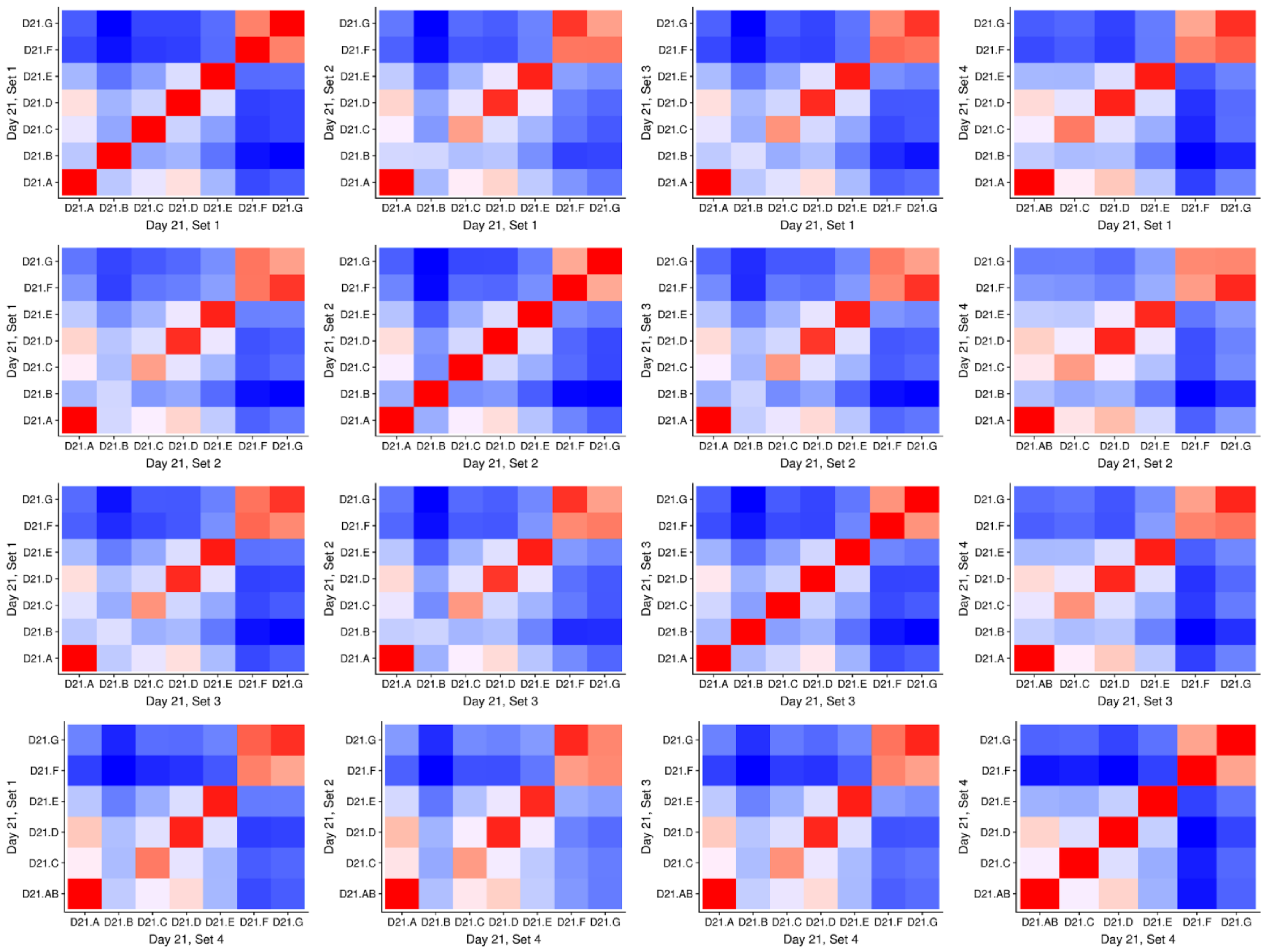

Supplemental Figure 6. Intra-day centroid correlation for the four replicas of Day 21. Clusters correspond well across replicates of each timepoint consistent with no batch effect artifacts.

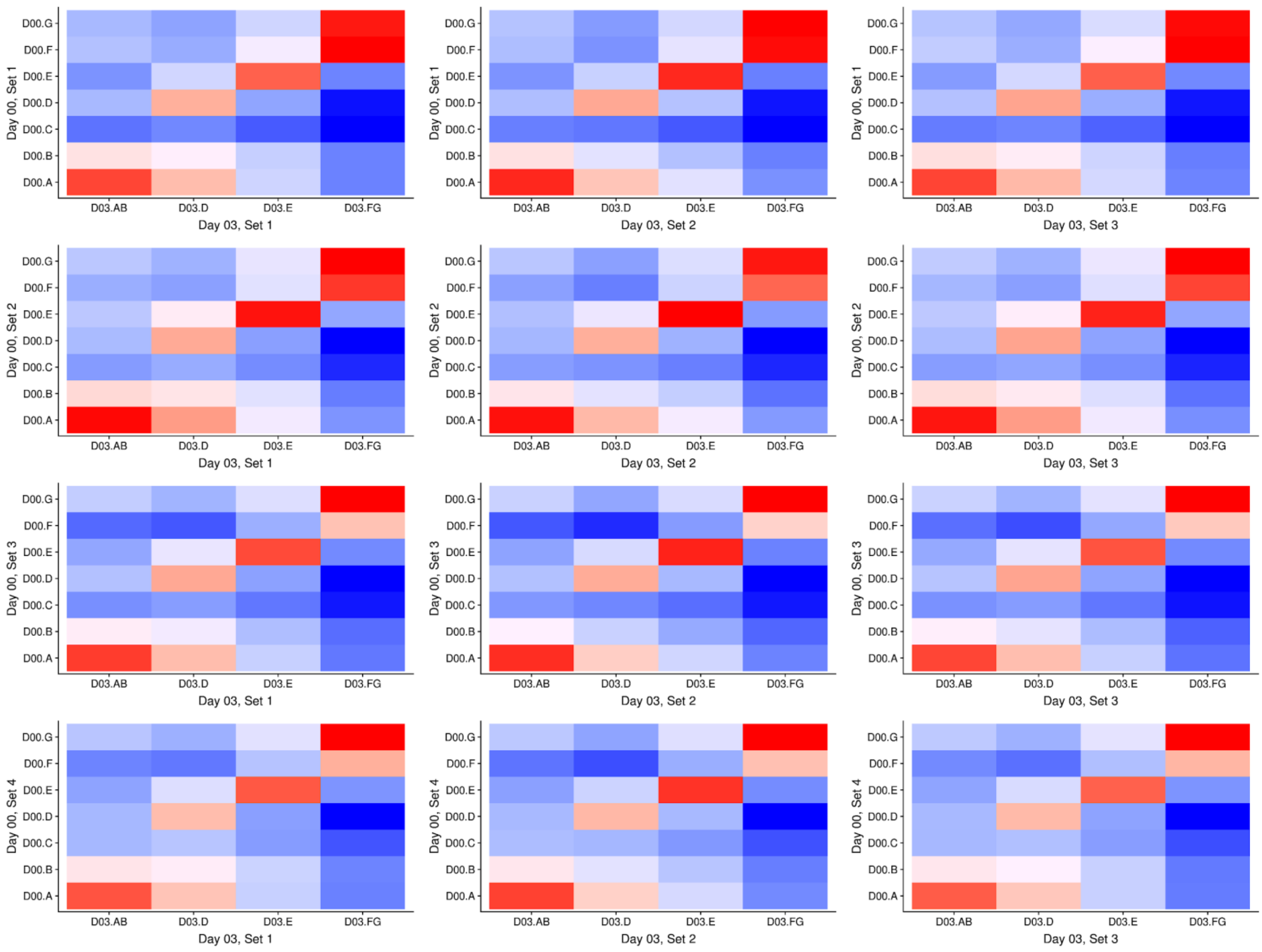

Supplemental Figure 7. Centroid correlation of Day 03 clusters and Day 00 clusters. Shared clusters suggest some cells seen at baseline are also seen shortly after burn tenotomy injury.

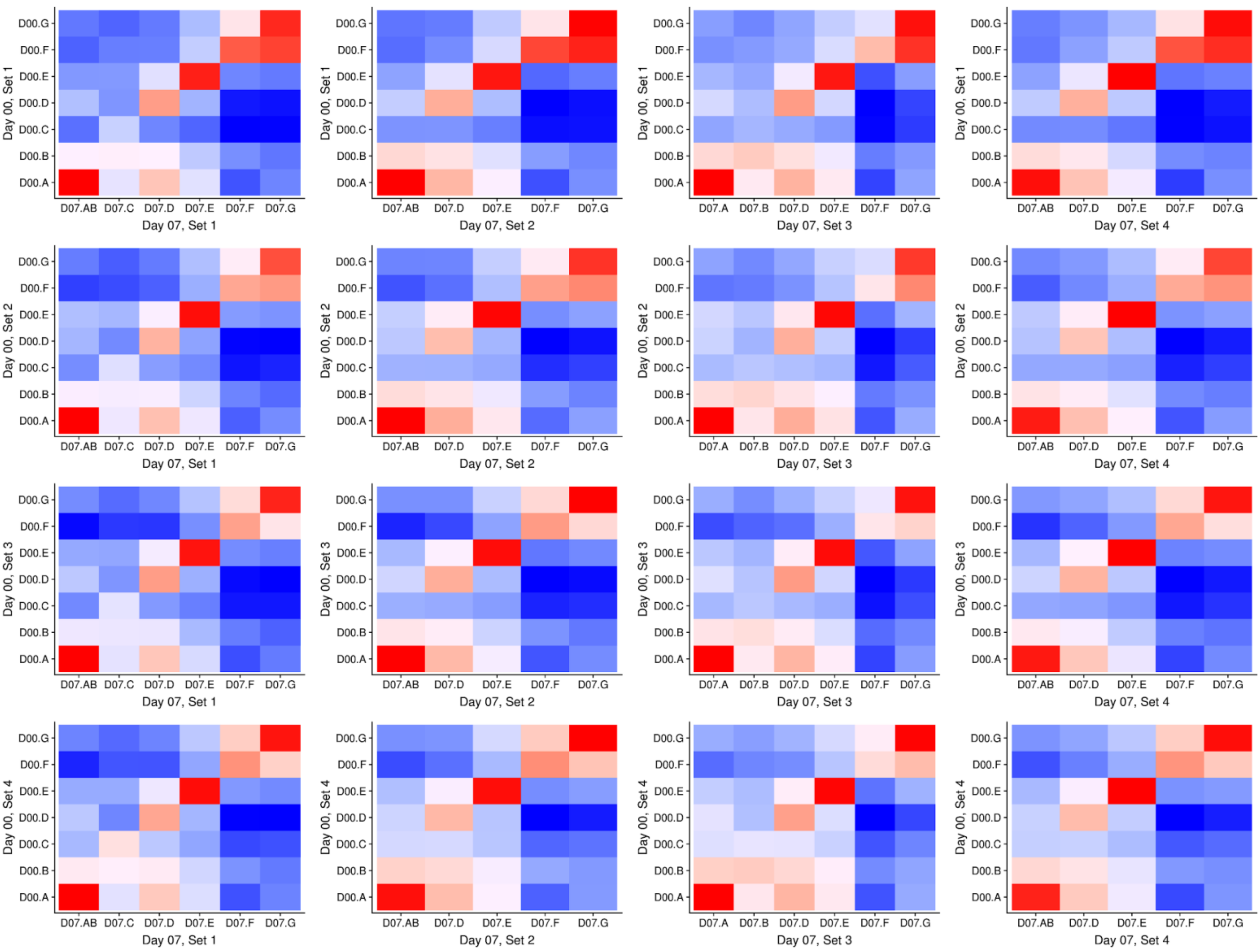

Supplemental Figure 8. Centroid correlation of Day 07 clusters and Day 00 clusters demonstrating corresponding cell populations between baseline and post-injury date indicated.

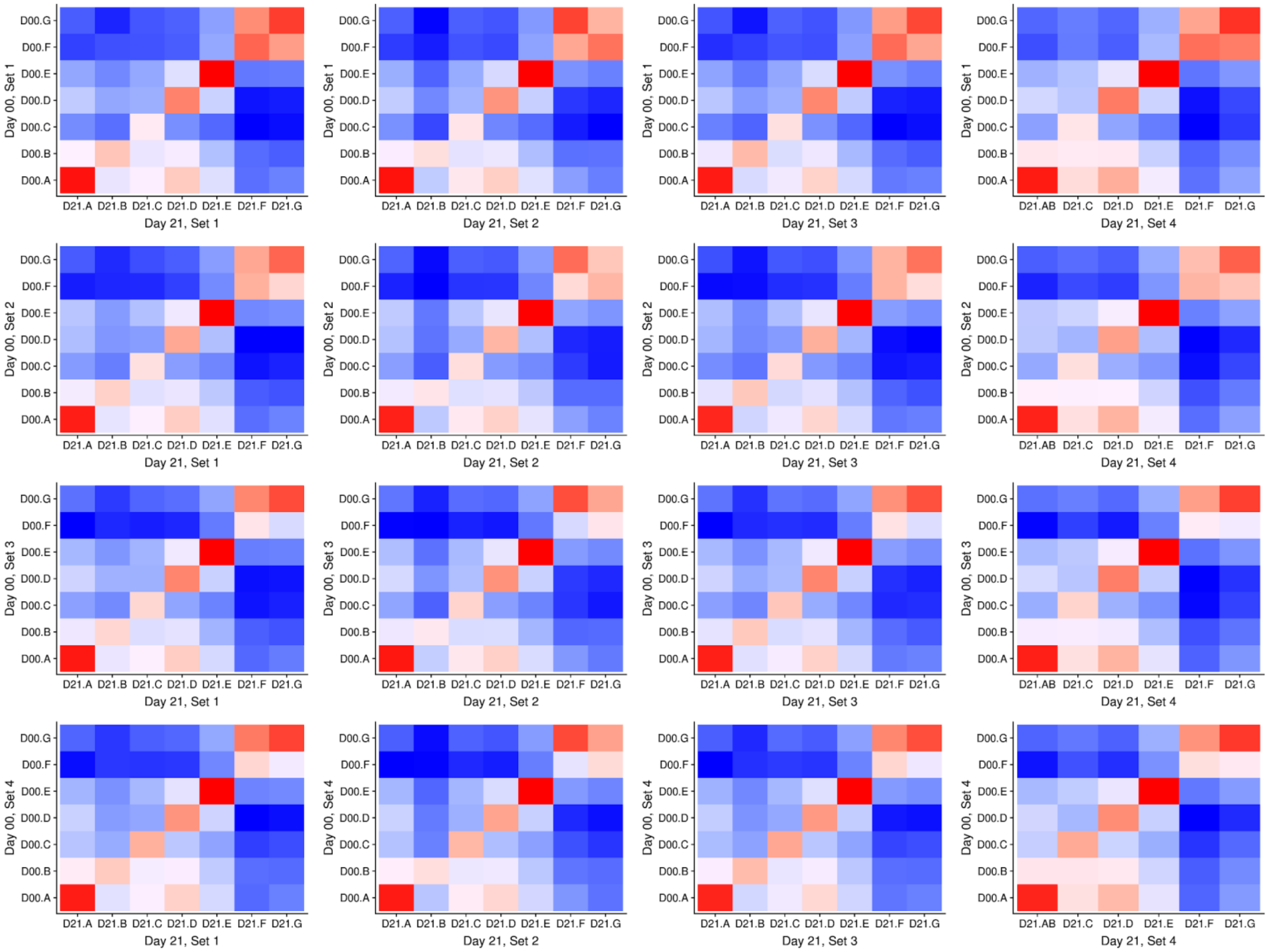

Supplemental Figure 9. Centroid correlation of Day 21 clusters and Day 00 clusters demonstrating corresponding cell populations between baseline and post-injury date indicated.

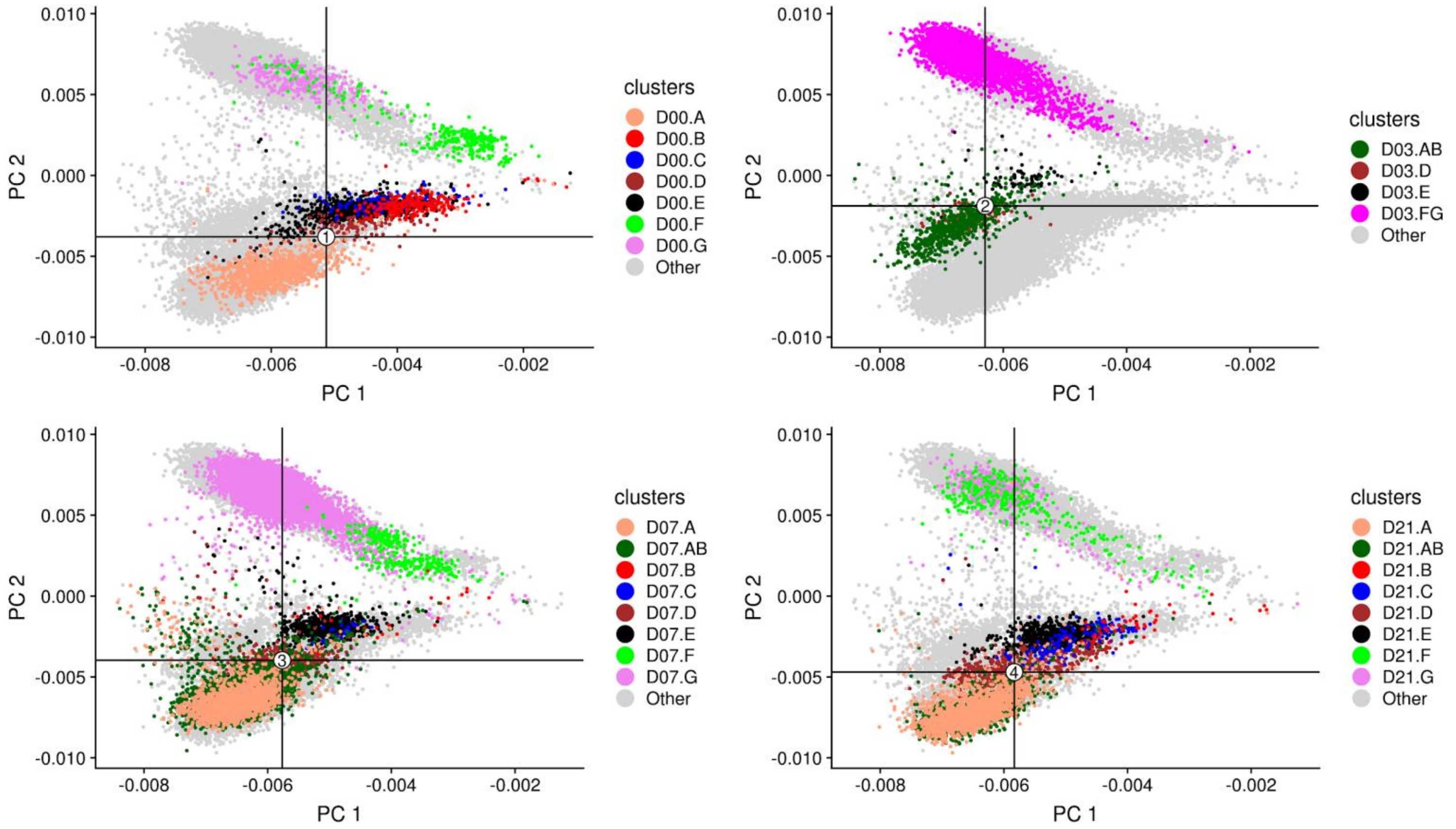

Supplemental Figure 10. PCA projection of the merged set from all time points, before the correction for the batch effect. Each panels shows the cells from a single time point, colored by provisonal cluster. The white circle is the mean of the centroids of A/AB, D, and E for each time point, now disaligned.

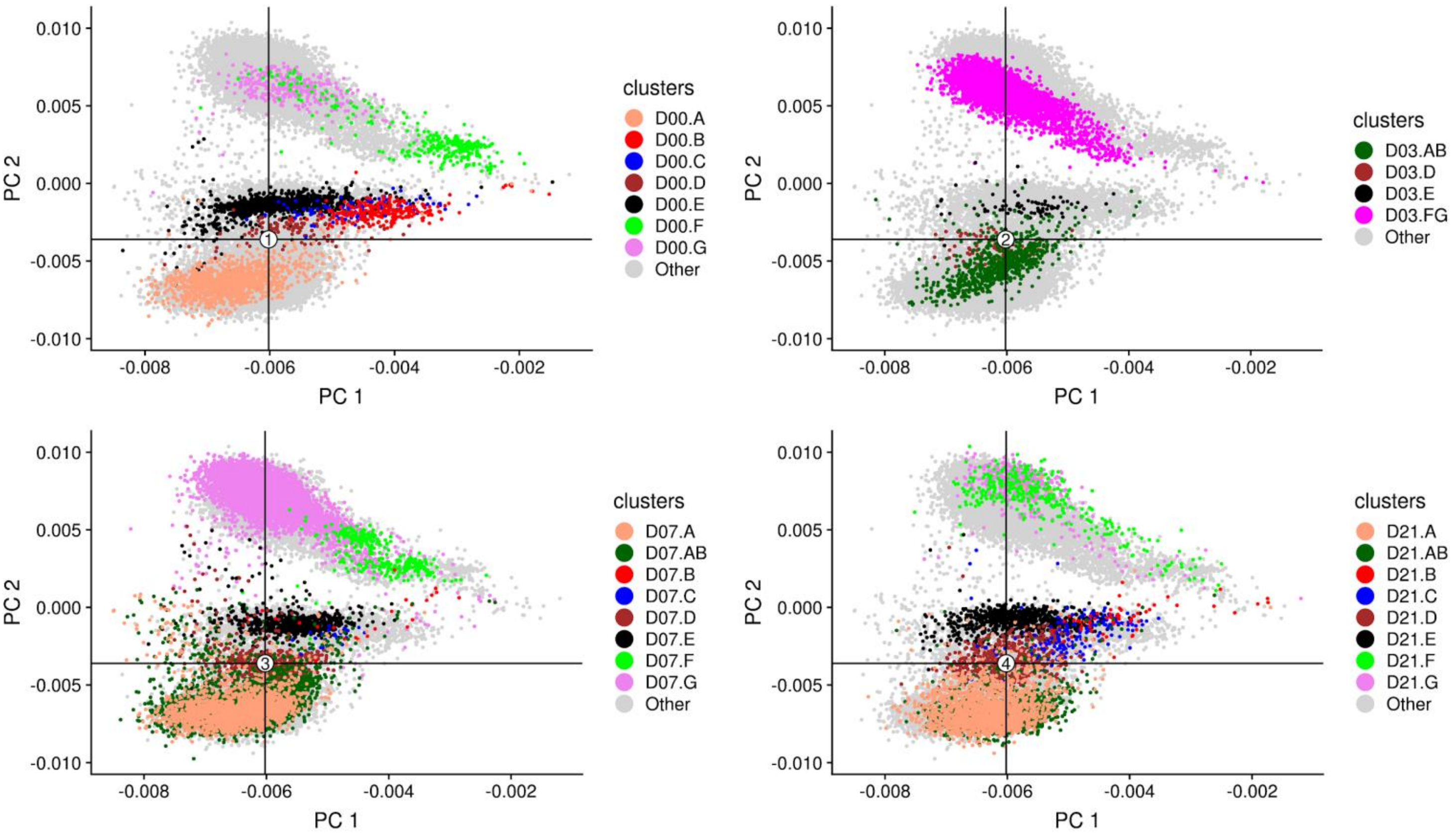

Supplemental Figure 11. PCA projection of the merged set from all time points, after the correction for the batch effect. Each panels shows the cells from a single time point, colored by provisional cluster. The white circle is the mean of the centroids of A/AB, D, and E for each time point, that are now aligned.

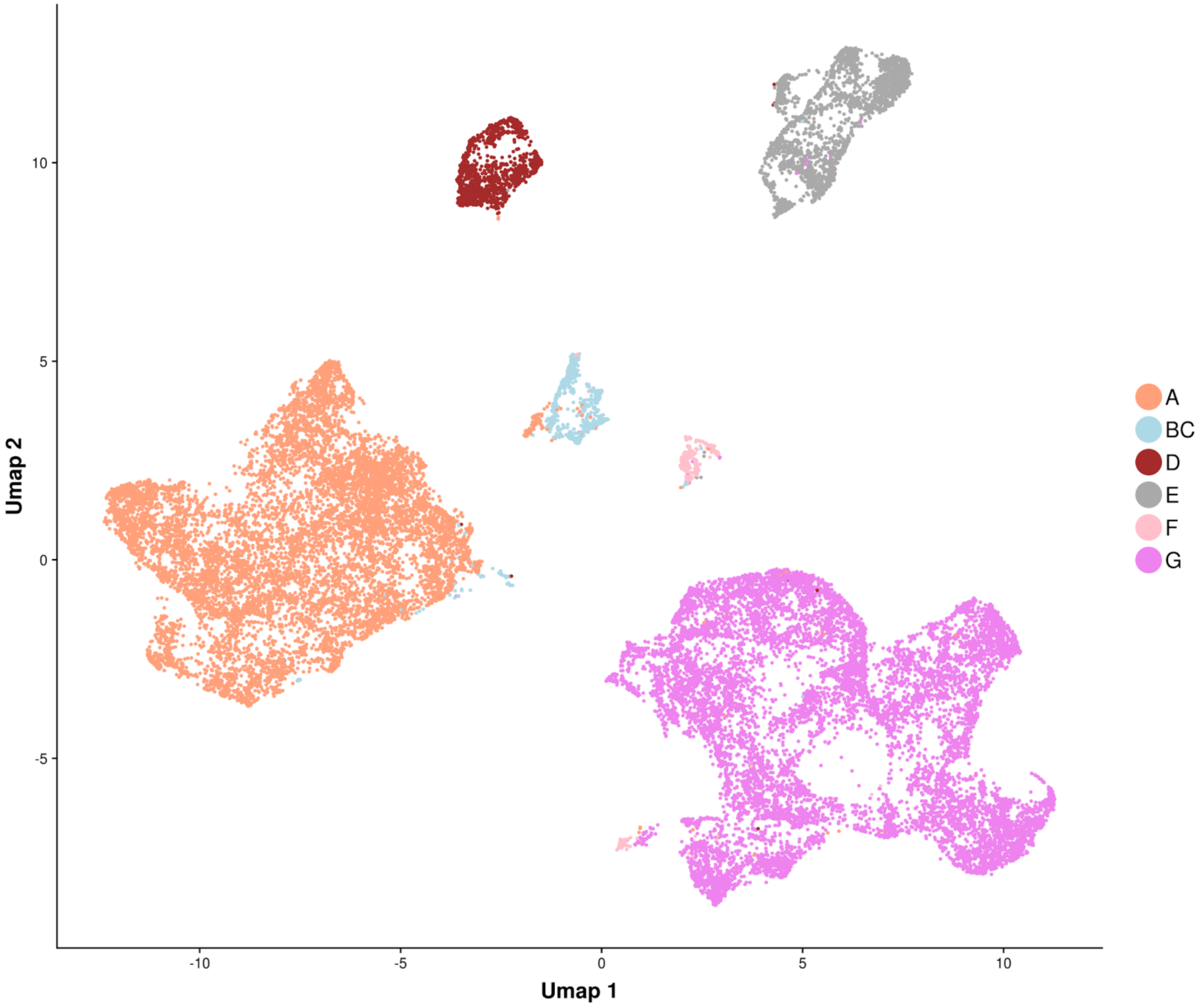

Supplemental Figure 12. UMAP projection of the merged set from all time points. Unsupervised clustering defined the final clusters A-G, however, clusters B and C are not separated.

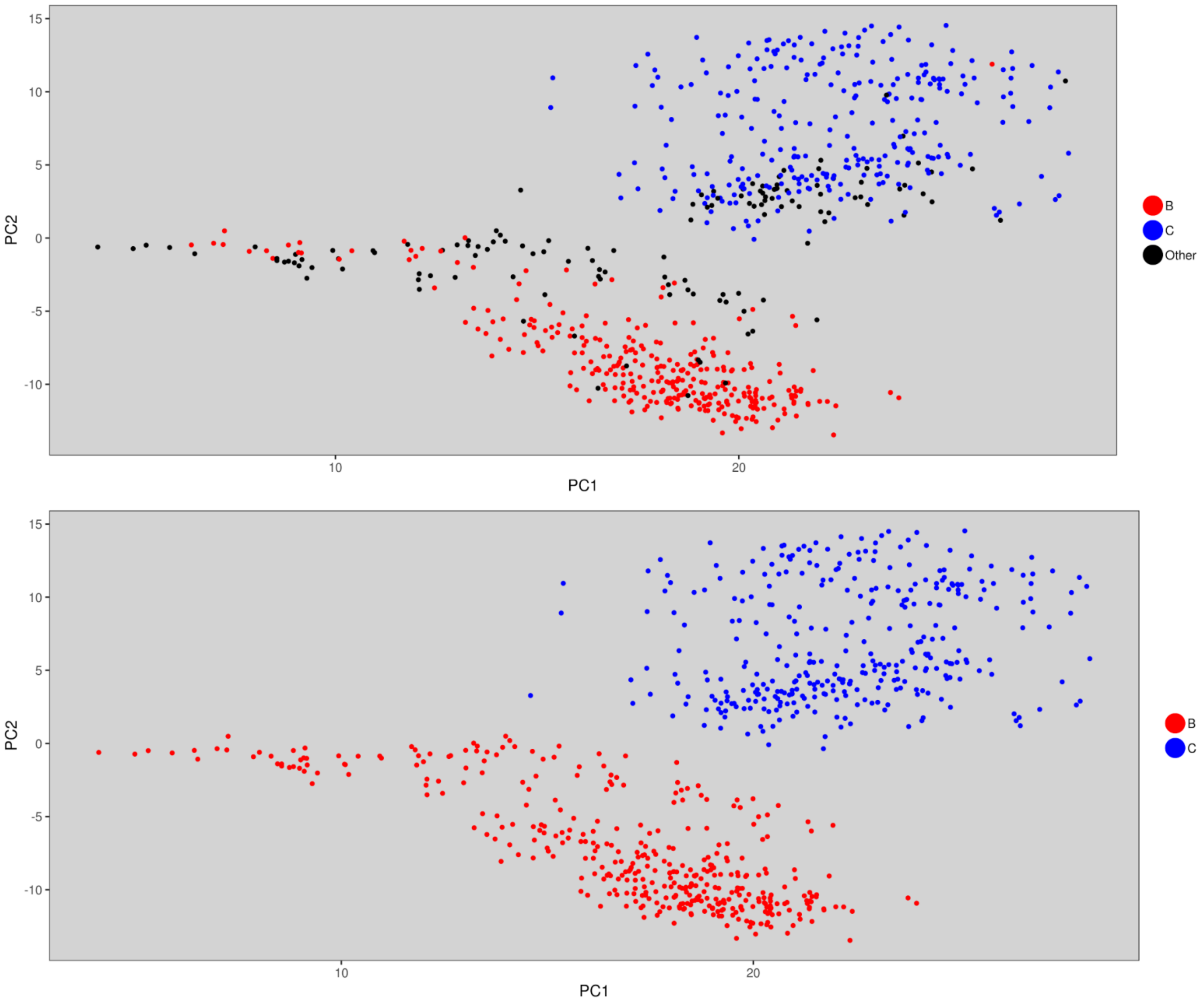

Supplemental Figure 13. PCA projection of BC. Top: provisional cluster attribution from centroid correlation analysis. Bottom: final cluster attribution after unsupervised clustering.

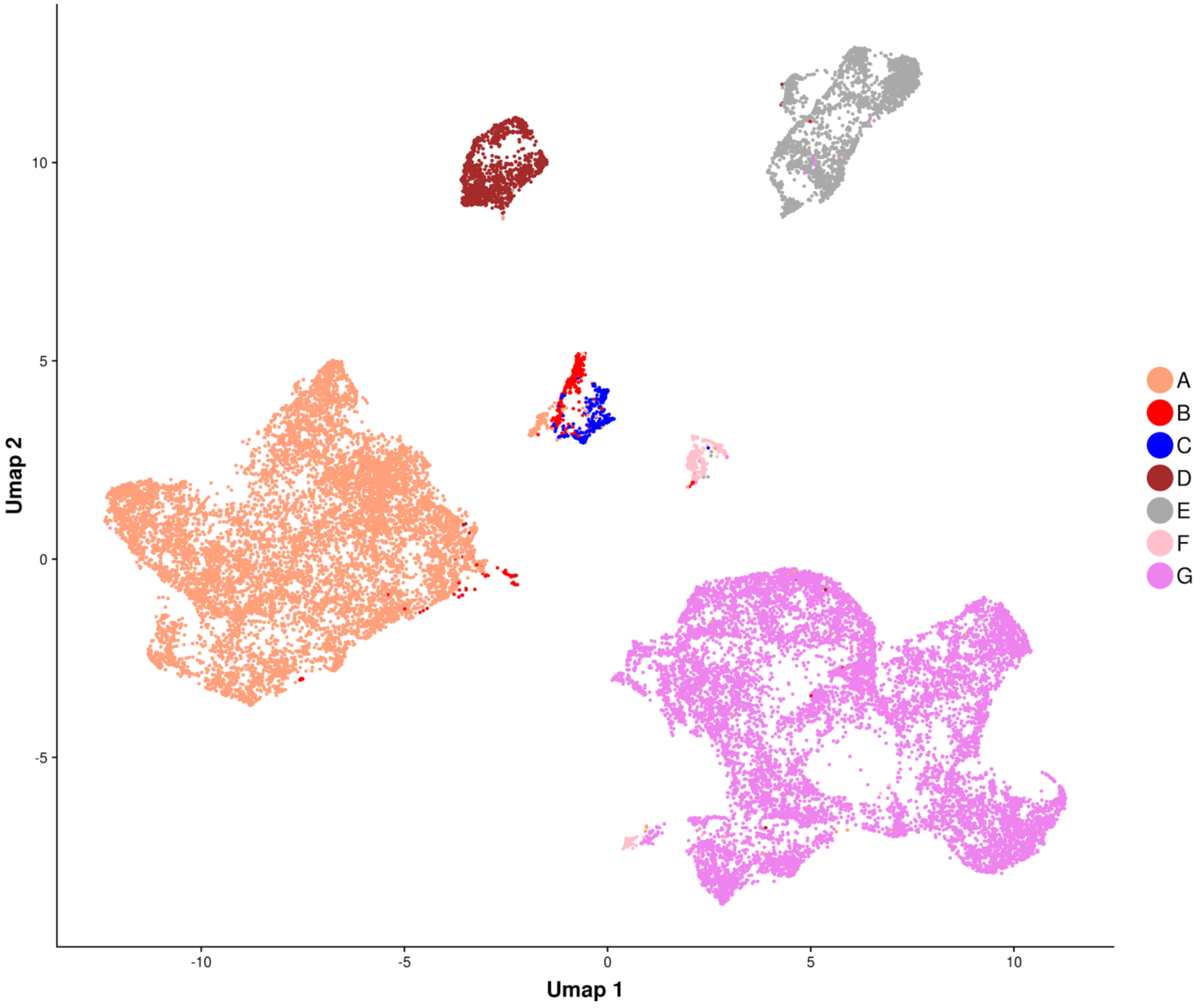

Supplemental Figure 14. UMAP projection of final clusters obtained by pulling cells from all the time points together

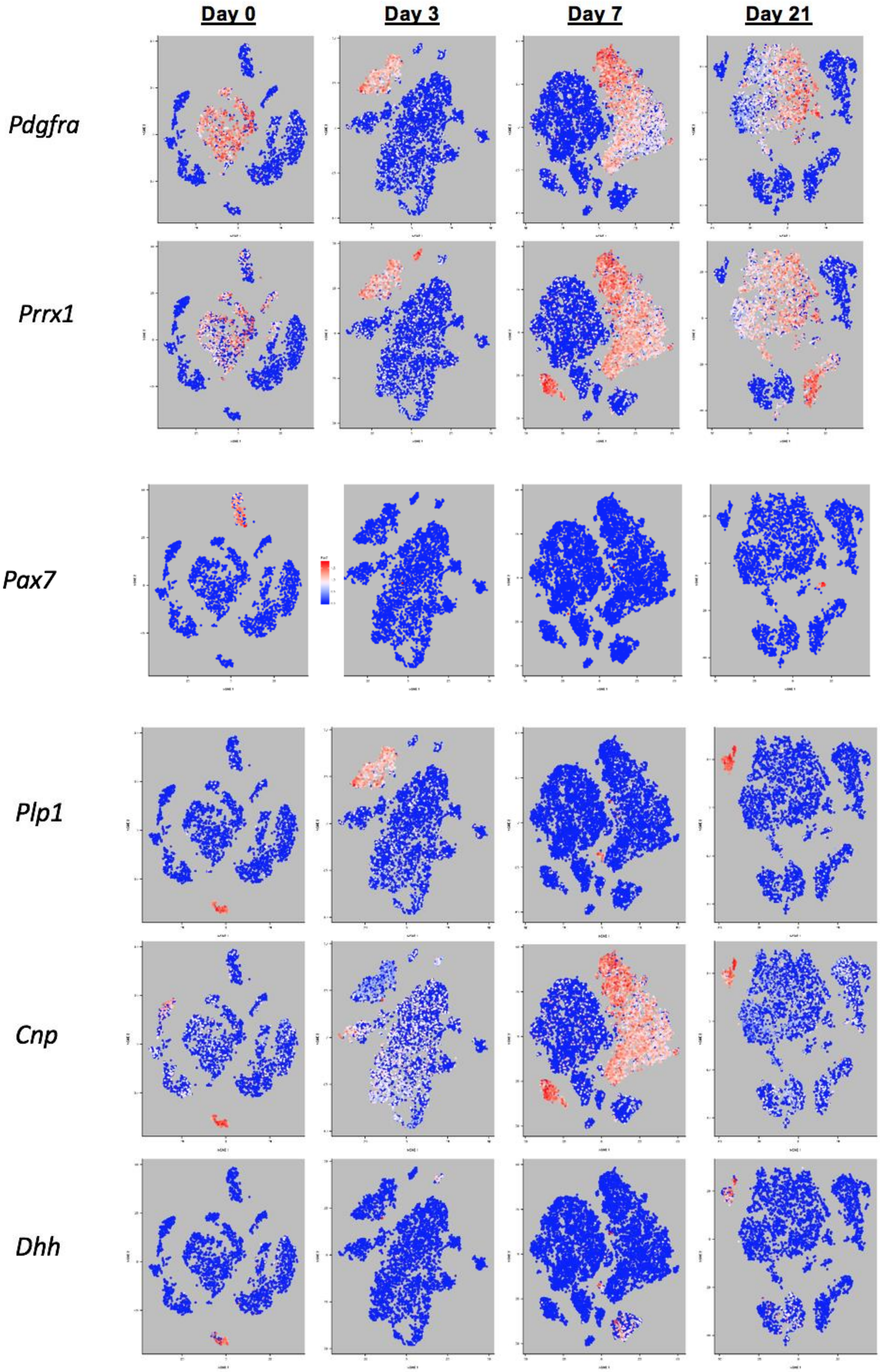
Day 0
Day 3
Day 7
Day 21
Pdgfra
Prrx1
Pax7
Plp1
Cnp
Dhh

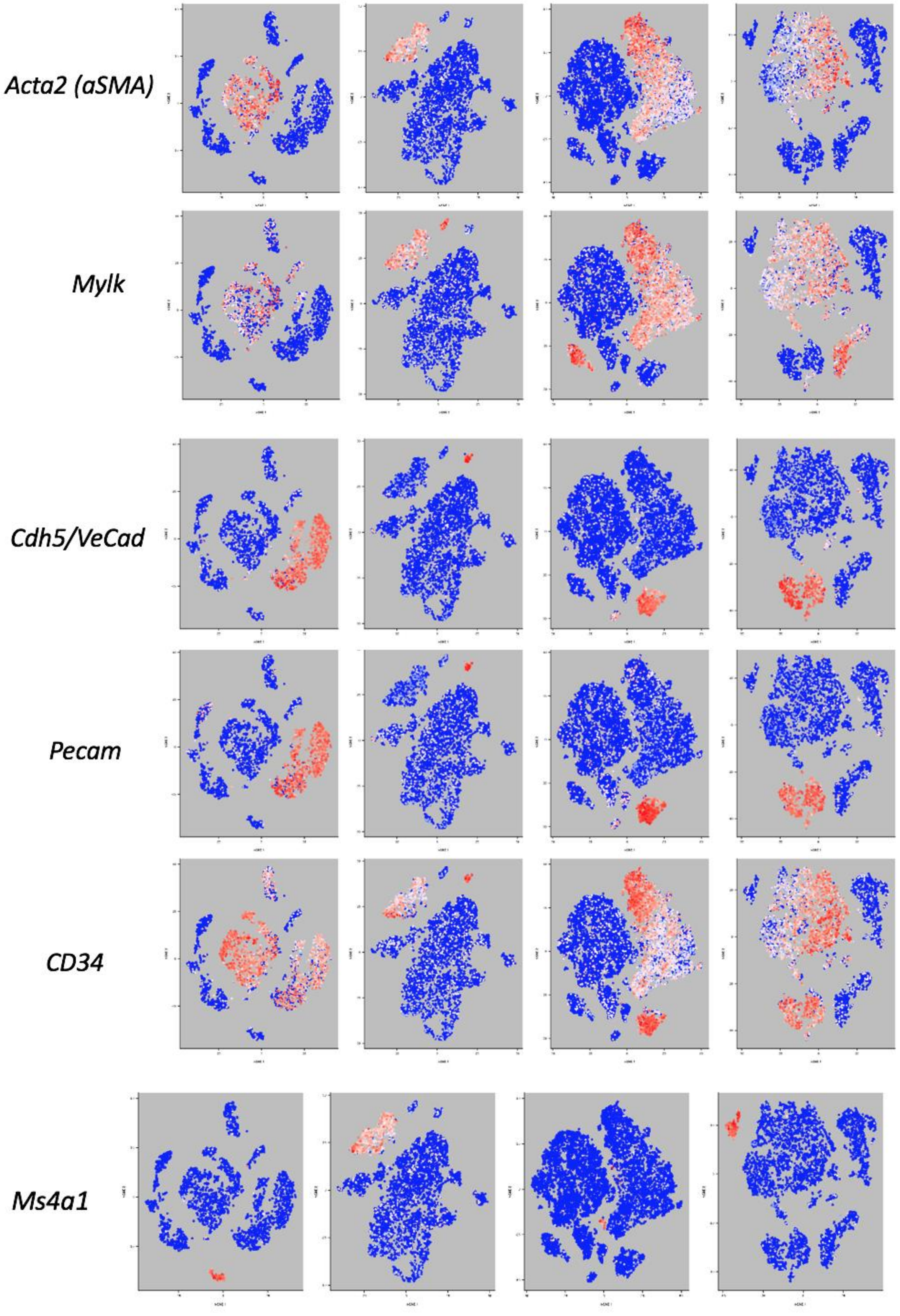
Acta2 (aSMA)
Mylk
Cdh5/VeCad
Pecam
CD34
Ms4a1

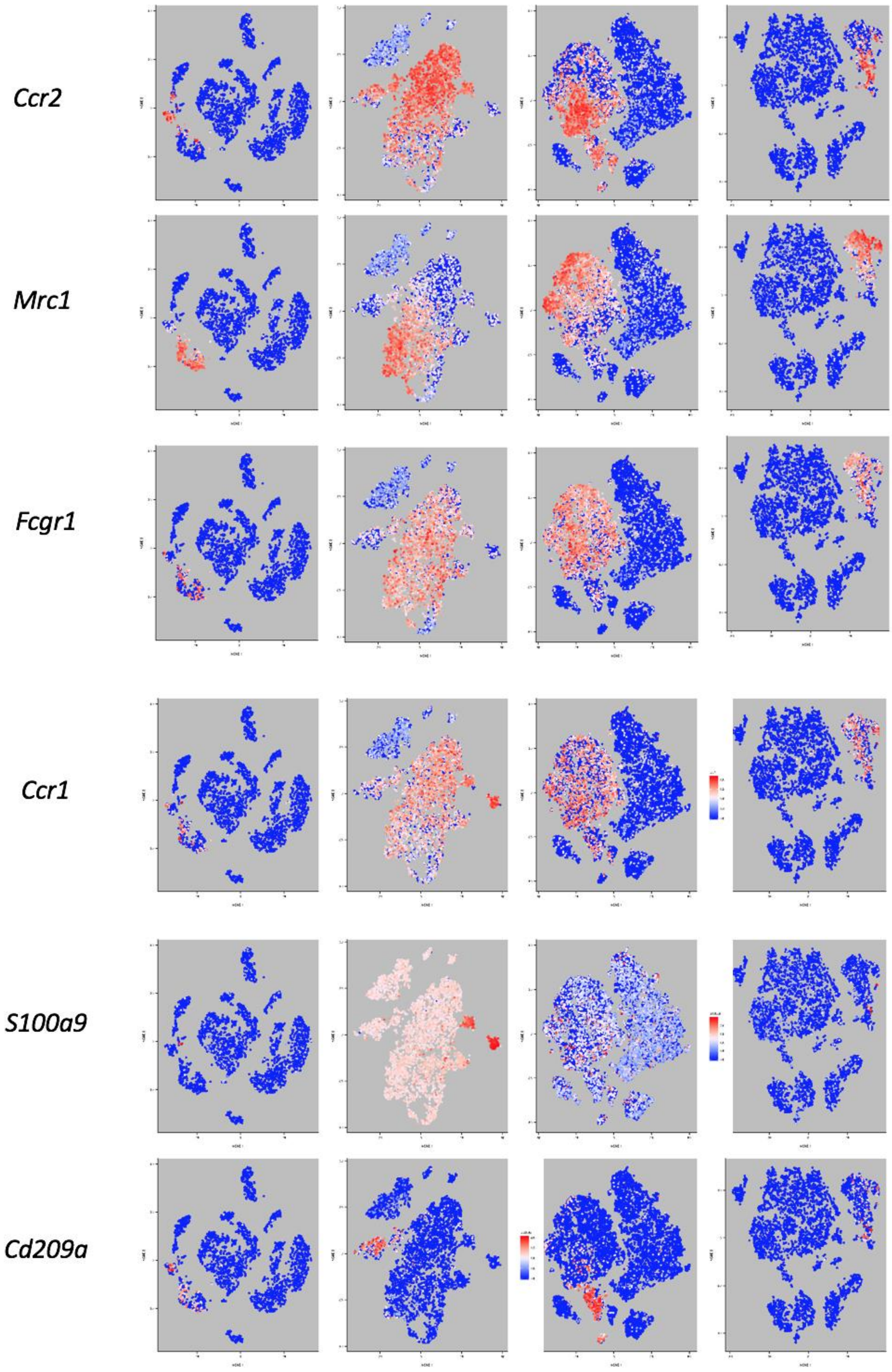

Supplemental Figure 15. Expression profile featureplots for characteristic cluster defining genes shown above (corresponding to genes reported in table 8)

Provisional \ Final	A	B	C	D	E	F	G
A	4656	15	18	6	2	1	3
AB	5766	33	35	13	2	0	0
B	3	307	1	1	0	2	0
C	13	0	285	0	0	0	0
D	4	0	0	1263	5	0	1
E	8	6	0	5	2514	0	13
F	4	0	0	1	0	407	512
FG	0	2	0	0	0	23	4650
G	12	20	3	3	0	61	6506

Supplementary Table 1. Cell numerosity in provisional and final clusters demonstrating that combined provisional clusters e.g. AB or FG were predominantly one type (A and G respectively).

Markers and enrichment

Markers were obtained by the Seurat function FindMarkers, and subsequently enriched with LRPath (under/over expression was determined by the logFC) [2].

Supplementary bibliography

[1] Butler, Andrew, et al. "Integrating single-cell transcriptomic data across different conditions, technologies, and species." Nature biotechnology 36.5 (2018)
[2] Kim JH, et al. “LRpath analysis reveals common pathways dysregulated via DNA methylation across cancer types.” BMC Genomics, 526.13 (2012)
